# Comprehensive transcriptome and metabolome profiling reveal metabolic mechanisms of *Nitraria sibirica* Pall. to salt stress

**DOI:** 10.1038/s41598-021-92317-6

**Published:** 2021-06-18

**Authors:** Huanyong Li, Xiaoqian Tang, Xiuyan Yang, Huaxin Zhang

**Affiliations:** 1 Research Institute of Pomology of Tianjin Academy of Agricultural Sciences, Tianjin, China; 2 Research Center of Saline and Alkali Land of National of Forestry and Grassland Administration, CAF, Beijing, China

**Keywords:** Plant stress responses, Salt

## Abstract

*Nitraria sibirica* Pall., a typical halophyte that can survive under extreme drought conditions and in saline-alkali environments, exhibits strong salt tolerance and environmental adaptability. Understanding the mechanism of molecular and physiological metabolic response to salt stress of plant will better promote the cultivation and use of halophytes. To explore the mechanism of molecular and physiological metabolic of *N. sibirica* response to salt stress, two-month-old seedlings were treated with 0, 100, and 400 mM NaCl. The results showed that the differentially expressed genes between 100 and 400 mmol L^−1^ NaCl and unsalted treatment showed significant enrichment in GO terms such as binding, cell wall, extemal encapsulating structure, extracellular region and nucleotide binding. KEGG enrichment analysis found that NaCl treatment had a significant effect on the metabolic pathways in *N. sibirica* leaves, which mainly including plant-pathogen interaction, amino acid metabolism of the beta alanine, arginine, proline and glycine metabolism, carbon metabolism of glycolysis, gluconeogenesis, galactose, starch and sucrose metabolism, plant hormone signal transduction and spliceosome. Metabolomics analysis found that the differential metabolites between the unsalted treatment and the NaCl treatment are mainly amino acids (proline, aspartic acid, methionine, etc.), organic acids (oxaloacetic acid, fumaric acid, nicotinic acid, etc.) and polyhydric alcohols (inositol, ribitol, etc.), etc. KEGG annotation and enrichment analysis showed that 100 mmol L^−1^ NaCl treatment had a greater effect on the sulfur metabolism, cysteine and methionine metabolism in *N. sibirica* leaves, while various amino acid metabolism, TCA cycle, photosynthetic carbon fixation and sulfur metabolism and other metabolic pathways have been significantly affected by 400 mmol L^−1^ NaCl treatment. Correlation analysis of differential genes in transcriptome and differential metabolites in metabolome have found that the genes of *AMY2*, *BAM1*, *GPAT3*, *ASP1*, *CML38* and *RPL4* and the metabolites of L-cysteine, proline, 4-aminobutyric acid and oxaloacetate played an important role in *N. sibirica* salt tolerance control. This is a further improvement of the salt tolerance mechanism of *N. sibirica*, and it will provide a theoretical basis and technical support for treatment of saline-alkali soil and the cultivation of halophytes.

## Introduction

Soil salinization has become a worldwide issue and environmental problem. In addition, salinity is a major adverse environmental factor that affects plant growth, crop productivity, microbial communities, and agricultural economics^1^. The Food and Agriculture Organization of the United Nations (FAO) has reported that more than 800 million ha of land worldwide are affected by salt^2^. Reasonable development and utilization of these vast saline soils could alleviate land resource problems; this alleviation is especially important with respect to the increasing demands associated with increasing global population. Researching salt-tolerant plants based on the understanding of the effects of salinity on both plant biochemistry and plant adaptation mechanisms is a formidable approach to solving this problem^3^.

Salinity has an immediate effect on seed germination, plant development and growth; these effects are due mainly to ion toxicity, osmotic stress and secondary oxidative stress^4,5^. Salt outside of roots can impose osmotic stress, which reduces the ability of plants to take up water; this stress leads to the immediate accumulation of reactive oxygen species (ROS), and the over-accumulation of Na^+^ and Cl^−^ leads to ionic stress, which affects photosynthetic machinery, plasma membrane integrity, and cellular metabolism^6^.

Plant adaptation to salinity occurs mainly via salt tolerance and salt avoidance. The salt stress adaptation or tolerance mechanisms used by plants include mainly tissue tolerance to osmotic stress, ion homeostasis and detoxification^6^. Plants maintain osmotic balance mainly by accumulating high concentrations of osmolytes in the cytoplasm. The accumulation of osmolytes can re-establish cellular redox balance by regulating the ROS content induced by salinity^7^. Osmolytes are small-molecular-weight organic compounds in plant cells; osmolytes mainly consist of sugars, polyhydric alcohols, and N-containing compounds^8–10^. Many genes and proteins that are salt stress responsive and that regulate the synthesis of osmolytes, including Δ1-pyrroline-5-carboxylase synthase (*P5CS*), mannitol-1-phosphate dehydrogenase (*mt1D*), and betaine aldehyde dehydrogenase (*BADH*), have recently been identified^11–14^.

Transcriptomics, proteomics, and bioinformatics have markedly increased our understanding of global plant system responses and adaptations to salt stress conditions^15^. Transcriptomics involves analysis at the mRNA level, but a transcriptome does not represent the complete protein expression of a proteome; in addition, translated proteins might not be enzymatically active^16,17^.

RNA-Seq technology has been applied in transcriptomic sequencing of various plants, such as *Brassica napus* L*.*, *Oryza latifolia* Desv., *Hordeum vulgare* L*.*, *Caragana korshinskii* Kom., etc.^18–21^. Many salt-tolerant genes related to secondary metabolism, ion absorption and transport, redox reactions, and control of cell and tissue growth were found by analyzing plant gene changes under salt stress^10^. The transcription level of plants changes under adverse conditions, and there are mainly two types of genes involved in the response to stress. One type of gene mainly encodes related protein genes that control the formation of metabolites, and maintain the balance of metabolism and osmosis. The other type encodes proteins related to signal receptors and transduction to ensure normal signal transduction in plants^22^. Many genes of these plants are induced that either directly protect the plant from salt stress or regulate the expression of other target genes upon exposure to salt stress^23^. It has been found by transcriptomics that the genes *PtSOS2*, *NHXs* and *HKTs* have been observed to play an important role in improving plant salt tolerance by controlling ion balance through ion transport^24–26^. Studying the transcriptome and exploiting different salt tolerance genes are greatly important for understanding the mechanisms of plant salinity tolerance.

Metabolomics involves the comprehensive analysis of plant metabolites and secondary metabolism. In particular, metabolomics can help reveal the complex mechanisms governing the relations between plants and the environment; these relations are analyzed by studying the relationships among metabolic networks, metabolic regulation, phenotypes and plant growth^27,28^. Hence, metabolomics, as a functional genomics methodology, has been applied both to study the complex molecular interactions in biological systems under salt stress and as a feasible option for the biotechnological improvement of halophytes^29^.

Current technological methods, including gas chromatography-mass spectrometry (GC–MS), liquid chromatography-mass spectrometry (LC–MS), nuclear magnetic resonance (NMR), and Fourier transform ion cyclotron resonance-mass spectrometry (FTICR-MS), have been widely used to analyze plant metabolism^30,31^. Many abiotic stress-related metabolomic studies have recently been performed. Metabolic changes in *Arabidopsis thaliana* in response to temperature stress, salt stress, K nutrition, and heat and drought stress combined under different conditions have been studied using metabolomics^32–35^. Moreover, metabolic fingerprinting of salt stress responses has been performed for tomato, soybean, barley, *Casuarina glauca*, *Aeluropus lagopoides*, wheat and other plants^36–41^. The qualitative and quantitative analyses of metabolites such as sugars, sugar alcohols, amino acids, organic acids and polyamines can reveal plant biomarkers and metabolic regulatory network responses to abiotic stress. Proline, putrescine, spermidine and spermine contents are positively correlated with plant resistance to abiotic stress^42–45^. With the exception of a report on metabolic profile changes in *Nitraria tangutorum* Bobr. suspension cells under salt stress, no relevant metabolomic research on *N. sibirica* has been reported thus far^46^. The metabolic profiles of suspension cells are somewhat limiting, as the profiles cannot explain the salt-tolerant mechanisms of whole plants.

*Nitraria sibirica* Pall. (*N. sibirica*) is a shrub that can survive under extreme drought and in saline-alkali environments. *N. sibirica* is a typical woody salt-diluting halophyte and exhibits strong salt tolerance and environmental adaptability^47–49^. Thus, the salt tolerance mechanisms of *N. sibirica*, including its osmotic adjustment, distribution of Na and K, and changes in oxidase activity, have been increasingly studied^50–53^. Many salt tolerance genes of *Nitraria* L., including *NsNHX1*, *NtNHX1*, *NtP5CS* and *NtCIPK2*, have been cloned and functionally analyzed^54–59^. In our previous study, we characterized the transcriptome, gene expression profile and ionic responses of *N. sibirica* under salt stress^60^.

The roles of physiological index and several key regulatory genes involved in salt tolerance of *N. sibirica* have been examined; however, only these limited indicators cannot scientifically evaluate the salt tolerance of *N. sibirica*, the molecular and metabolic global regulatory networks response to salt stress at the transcriptome and metabolome level are still poorly understood. In this study, Illumina sequencing and gas chromatography-time-of-flight-mass spectrometry (GC-TOF–MS) were used to analyze the differentially expressed genes and metabolite profiles of *N. sibirica* involved in the salt tolerance. Those results allowed the identification of both salt-responsive genes and metabolites, and alterations to several metabolic pathways. This study facilitates to explore the regulation network of salt tolerance of *N. sibirica*, and provides a comprehensive understanding of plant salt tolerance mechanisms.

## Results

### Changes in soluble sugar, proline, amino acid and ABA contents in *N. sibirica* leaves under salt stress

**Figure 1:**
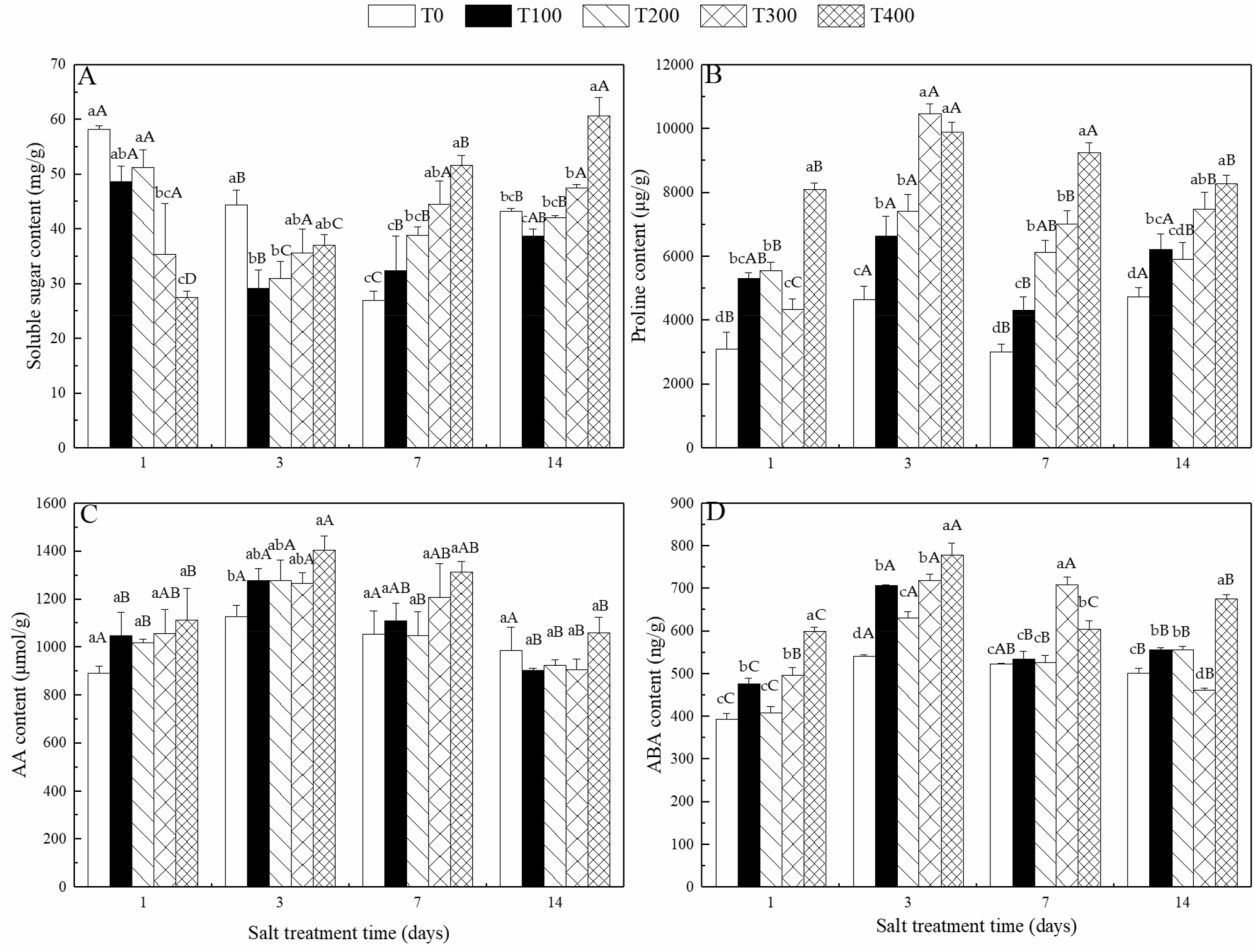
Soluble sugar (**A**), proline (**B**), amino acid (**C**) and ABA contents (**D**). Values represent the means ± SDs of three replicates, and the different small letters on histogram represent significant difference at *P* < 0.05 among different NaCl concentrations, while different capital letters represent significant difference at *P* < 0.05 among different treatment time in same NaCl concentrations.

The soluble sugar, proline, amino acid and ABA contents differed at different times after treatment, and the soluble sugar, proline, amino acid and ABA contents increased gradually as the treatment concentrations increased (Fig. 1). The leaves of *N. sibirica* seedlings treated with 400 mM NaCl for three days had a soluble sugar content of 7.94 mg g^−1^ fresh weight (FW); this content significantly differed from that of the control (Fig. 1A). The proline content was significantly altered by the different treatments. Compared with that in response to the control, the proline content clearly increased in response to treatment with 400 mM NaCl, and three days after treatment, the proline content remained at a high level (Fig. 1B). The amino acid content increased gradually as the treatment concentration increased. With the exception of seedings at three days after treatment, no significant differences between treatments were observed (Fig. 1C). The ABA content also significantly changed in response to different treatments and significantly increased in response to NaCl (Fig. 1D). A comprehensive analysis revealed that the osmotic substances and ABA contents in response to the 400 mM NaCl treatment significantly differed from those in response to the control treatment, but no significant differences were observed between the 100 mM NaCl treatment and the control treatment. Moreover, the osmotic substances and ABA contents peaked three days after treatment, after which they remained at high levels.

### Identification of differentially expressed genes (DEGs)

**Figure 2:**
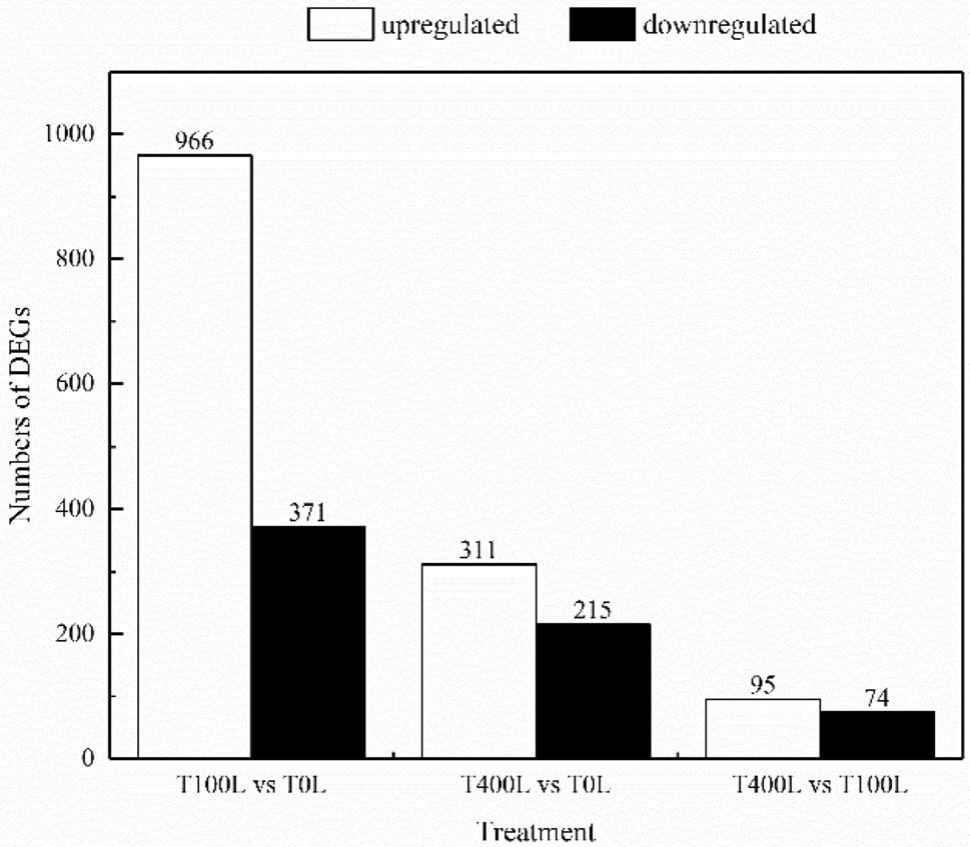
Number of DEGs in *N. sibirica* treated with different concentrations of NaCl.

In this study, DESeq software was used to screen differentially expressed genes (DEGs) between different samples. The DEGs between no salt (T0L), 100 mM NaCl (T100L), and 400 mM NaCl (T400L) treatments are shown in Fig. 2 and Fig. S1. There were 1337 DEGs between T100L-treated and T0L-treated leaves of *N. sibirica*, among which 966 were upregulated and 371 were downregulated. There were 526 DEGs between T400L-treated and T0L-treated leaves of *N. sibirica*, of which 311 were upregulated and 215 were downregulated. There were only 169 DEGs between T100L- and T400L-treated leaves of *N. sibirica*, of which 95 were upregulated and 74 were downregulated. Comparison of the DEGs between the T100L and T0L treatments to the DEGs between the T400L and T0L treatments revealed 197 common DEGs in leaves of *N. sibirica*, and comparison of the DEGs between the T400L and T0L treatments to the DEGs between the T400L and T100L treatments showed 25 common DEGs in leaves of *N. sibirica*. Analysis of different DEGs revealed that compared with no salt treatment, the number of DEGs induced by low-concentration NaCl treatment was significantly greater than high-concentration NaCl treatment.

### Gene ontology (GO) enrichment analysis of DEGs

**Figure 3:**
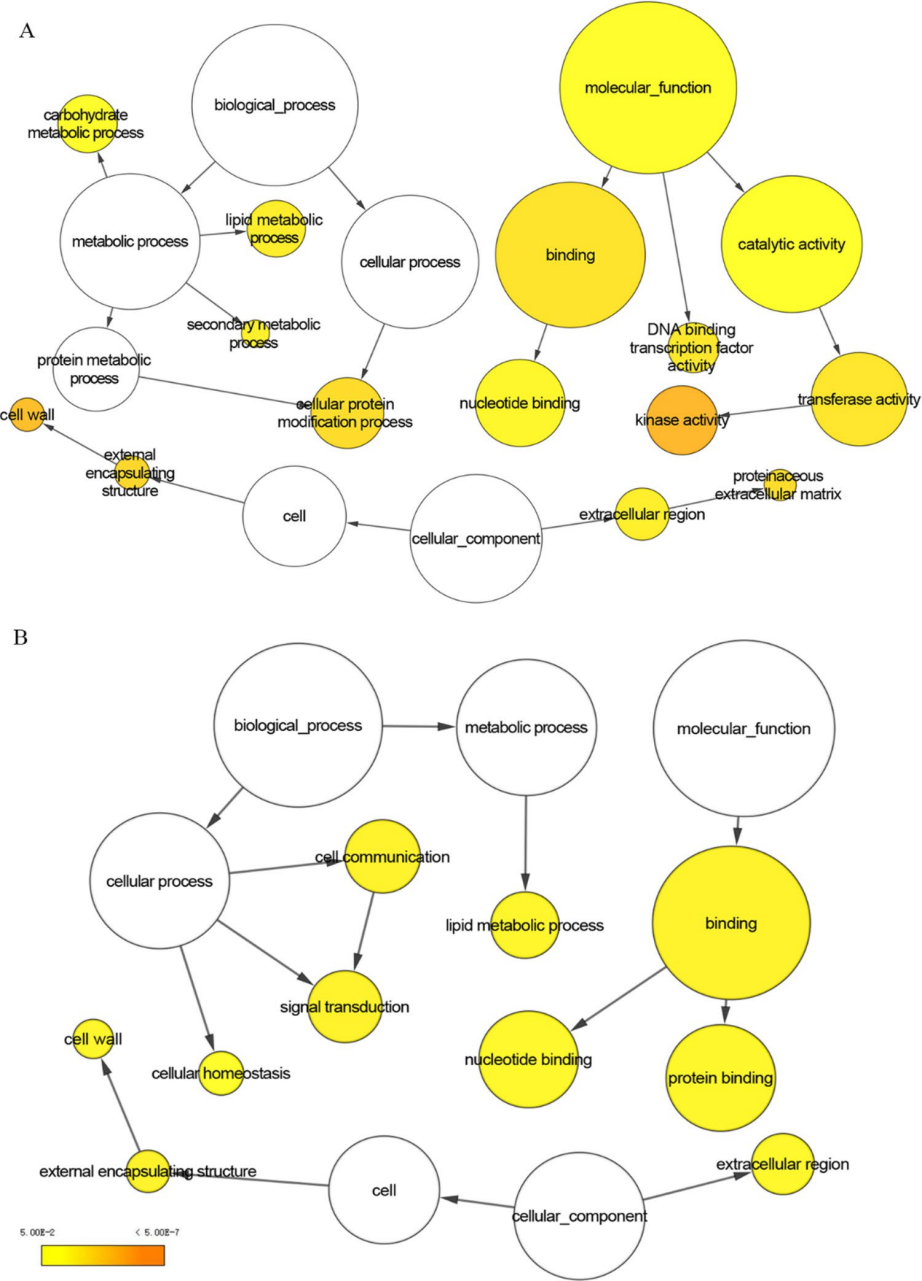
GO enrichment maps of DEGs under different NaCl treatments. (**A**), T100L vs T0L; (**B**) T400L vs T0L. Node size represents the gene number per node, and the colour of the node represents the *p*-value.

Expression analysis of DEGs between different samples can better reveal the changes in transcription level when the leaves of *N. sibirica* are treated with different concentrations of NaCl. The GO enrichment analysis of DEGs used GO-slim plant as the space name, and the significance output threshold P-value was set to 0.05. GO enrichment analysis of all DEGs was divided into three categories: biological processes, cellular components, and molecular function. As shown in Fig. 3, compared with T0L treatment, the T100L and T400L treatments were enriched most for unigenes of the binding term (GO: 0005488), with 603 and 257 DEGs, respectively, demonstrating significant enrichment. Compared with the T0L treatment, the two NaCl treatments were both enriched for many unigenes in cellular process (GO: 0009987), metabolic process (GO: 0008152), and catalytic activity (GO: 0003824), and the number of upregulated genes was greater than downregulated genes. After the two NaCl treatments, cell wall (GO: 0005618), external encapsulating structure (GO: 0030312), and extra cellular region (GO: 0005576) also demonstrated significant enrichment. DEGs between the T400L and T100L treatments were mainly enriched in terms of metabolic processes, binding, and cellular processes, while there were no significantly enriched terms between the two treatments.

### Kyoto encyclopedia of genes and genomes (KEGG) analysis of DEGs

KEGG analysis of all DEGs was performed using KOBAS 2.0 software. Figure S2 shows the metabolic pathways involving some DEGs. Under the T100L and T400L treatments, most DEGs were enriched in interactive metabolic pathways between plants and pathogens, with 27 and 7 DEGs, respectively. The up-regulated genes were mainly involved in the regulation of respiratory oxidative enzymes, calmodulin, *WRKY* transcription factors, and heat shock proteins, which are closely related to energy metabolism, signal transduction, and stress regulation. After treating *N. sibirica* with different concentrations of NaCl, β-alanine, arginine, proline, and glycine metabolism of amino acid metabolic pathways, and glycolysis, gluconeogenesis, galactose, starch, and sucrose metabolism of carbon metabolism pathways, were significantly changed in the leaves of *N. sibirica*. These metabolic pathways related to the synthesis and degradation of osmoregulatory substances underwent significant changes, which could not only provide energy sources for plant growth but also facilitate osmotic regulation through changes in metabolite contents, thereby improving the tolerance of *N. sibirica* to salt stress. After the NaCl treatments, plant hormone signal transduction pathways in the leaves of *N. sibirica* were enhanced, indicating that hormones play an important role in the salt tolerance of *N. sibirica*. Under the T100L and T400L treatments, the alternative splicing process in the leaves of *N. sibirica* comprised 10 and 4 unigenes, respectively, which could regulate the expression of genes related to the growth and development of plants and physiological processes related to salt tolerance to help the plants adapt to adverse environments.

### Verification of transcriptome data by quantitative polymerase chain reaction (qPCR)

To verify the accuracy of the transcriptome data of *N. sibirica*, 9 differentially expressed unigenes related to amino acid metabolism, glucose metabolism, transcription factors, and ion transport in the transcriptome data were selected for qPCR verification. As shown in Fig. S3, the verification results showed that the expression trends of the 9 unigenes were basically the same. There were some differences between the fold and transcriptome sequencing results due to differences in the detection platforms. Therefore, qPCR analysis confirmed that the transcriptome sequencing results for *N. sibirica* were accurate.

### Metabolic changes in response to salt stresses

In this study, the metabolic profiles of *N. sibirica* leaves were investigated. A definitively strong signal, a large peak capacity, and reproducible retention time were observed in all of the total ion chromatograms (TICs), indicating the reliability of the metabolomic analysis (Fig. S4). Totals of 619 effective peaks were detected, and a total 268 kinds of metabolites were identified in leaves (Table S1).

**Figure 4:**
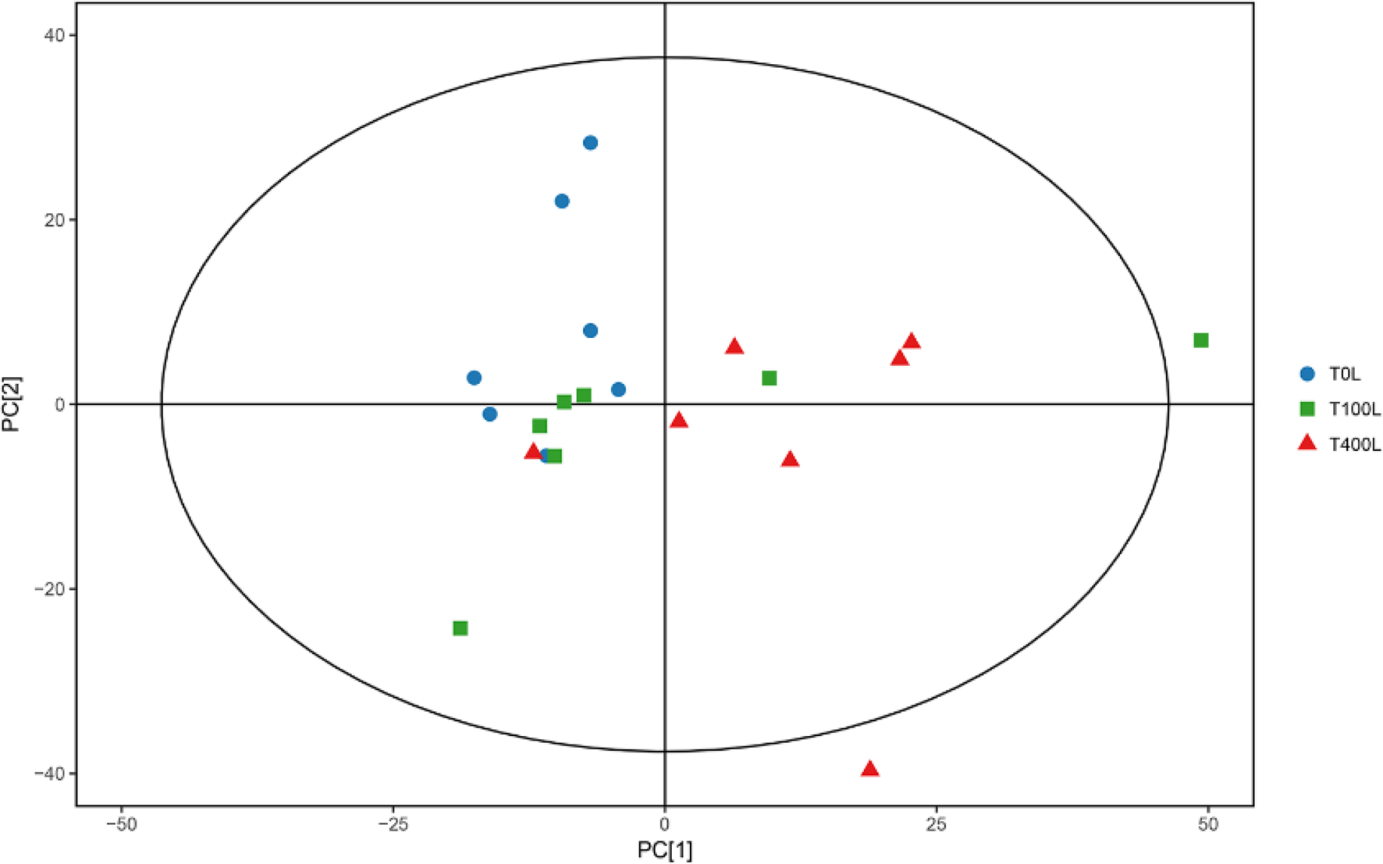
Principal component analysis (PCA) score plots of metabolic profiles in the leaves of seedlings under salt stress.

An unsupervised multivariate statistics-PCA and supervised multivariate statistics-orthogonal projections to latent structures-discriminate analysis (OPLS-DA) were performed by SIMCA 14.1 software. The results of the PCA showed that the metabolite responses were clearly separate between T0L and T400L treatments, whereas the responses were not clearly separate between T0L and T100L treatments (Fig. 4). Compared with the PCA, the OPLS-DA could filter the classification of the unrelated orthogonal variables of metabolites and analyze the non-orthogonal and orthogonal variables to obtain more reliable information concerning metabolite differences between groups and correlations among experimental groups^61^. The OPLS-DA score plots describing the distribution of the data are shown in Fig. S5; all groups were in the 95% Hotelling’s T-squared ellipse, and a clear distinction was visible between every two different experimental groups.

### Identification of salt-responsive metabolites in *N. sibirica*

**Figure 5:**
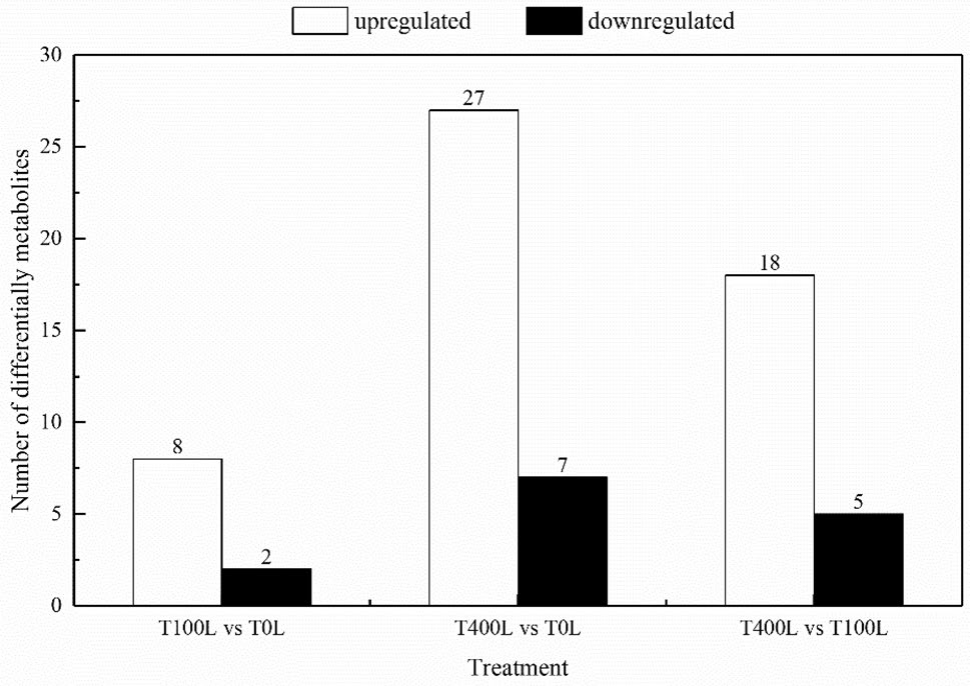
Number of differentially accumulating metabolites in the leaves of *N. sibirica* seedlings under salt stress.

To refine the prior analyses, the first principal component of VIP (VIP values exceeding 1.0) and the remaining variables assessed by Student’s t-test (*P*-value < 0.05) between two comparison groups were used to select differentially accumulating metabolites. Compared with those in response to the T0L treatment, the leaves of *N. sibirica* in response to T400L treatment presented 34 metabolites (27 upregulated metabolites and 7 downregulated metabolites), while only 10 metabolites (8 upregulated metabolites and 2 downregulated metabolites) in the leaves of *N. sibirica* differentially accumulated in response to T100L treatment (Fig. 5). The differentially accumulating metabolites mainly consisted of amino acids, sugars and polyols, and organic acids (Table S2).

**Figure 6:**
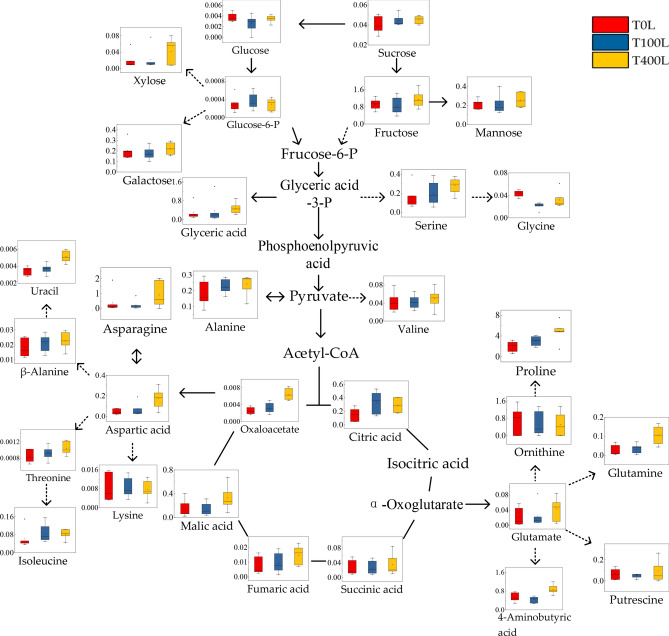
Changes in the metabolites of the metabolic pathways in the leaves of *N. sibirica* seedlings under salt stress.

The purported metabolites and the metabolic pathways of the tricarboxylic acid (TCA) cycle, glycolysis and amino acid synthesis in *N. sibirica* leaves under NaCl stress were simplified via a metabolic map to better understand the dynamically complex response mechanism to salt stress. Analysis of the metabolic map indicated that the amino acid (4-aminobutyric acid, alanine, asparagine, β-alanine, glutamine, glycine, proline, serine, valine, aspartic acid, threonine and isoleucine) and uracil contents in the leaves increased under T400L treatment, the content of alanine and serine increased by about one time compared with that of T0L treatment, while the content of proline and asparagine was more than two times that of T0L treatment. In the sugar metabolism pathway, the content of xylose, sucrose and mannose increased slightly, while the content of galactose, glucose, glucose-6-phosphate and other metabolites did not change significantly. After NaCl treatment, metabolites such as oxaloacetic acid, malic acid, fumaric acid, succinic acid and citric acid in the TCA cycle were significantly increased. Among them, the contents of citric acid and fumaric acid in the TCA cycle were about twice as high as those in the T0L treatment under the T400L treatment, and the contents of malic acid and oxaloacetic acid were about twice as high under the T400L treatment (Fig. 6).

### KEGG annotation of differentially accumulating metabolites

**Figure 7:**
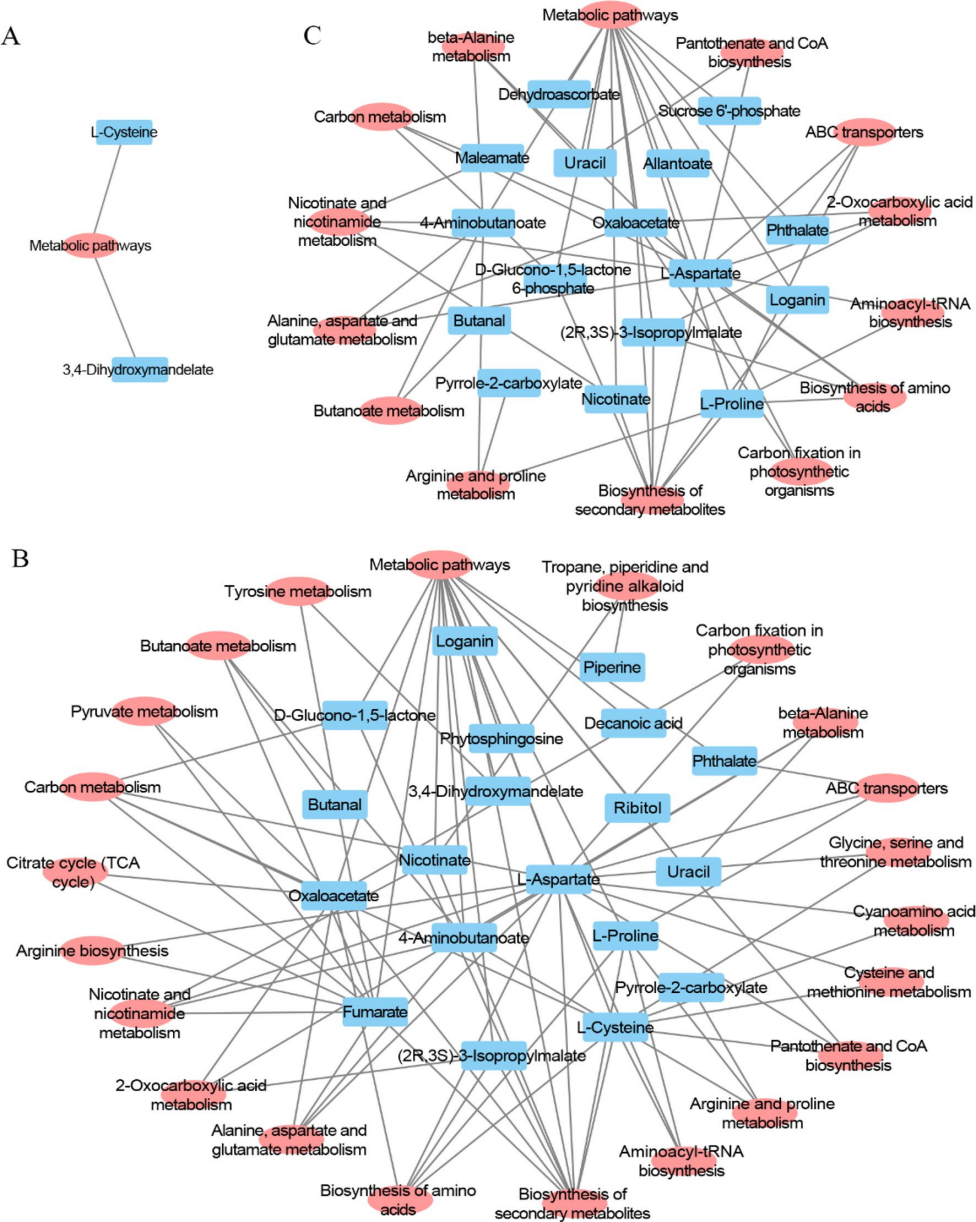
Metabolic pathways of differentially expressed metabolite mapped in *N. sibirica* leaves under different NaCl concentration treatment. Blue box shows the differentially expressed metabolite between different treatments, red oval shows the metabolic pathway of differentially expressed metabolite mapped. (**A**) T100L vs T0L; (**B**) T400L vs T0L; (**C**) T400L vs T100L.

The complex metabolic reactions and regulatory mechanisms that exist in organisms are not performed in isolation; different genes and proteins form complex pathways and networks that interact and regulate each other, leading to systemic changes in metabolic groups. The KEGG pathway database (www.kegg.jp/kegg/pathway.html) can be used to graphically demonstrate the biochemical processes in cells; these pathways are based on the functional information of genes and genomes and connections between possible metabolic pathways and their corresponding regulatory proteins^62,63^. All differentially accumulating metabolites that were affected by the salt stress treatments were mapped to *A. thaliana* (thale cress) in the KEGG pathway database. The results showed that many differential metabolites mapped to metabolic pathways. 2, 16 and 13 kinds of differential metabolites in the leaves were mapped to metabolic pathways in the comparisons of T100L vs. T0L, T400L vs. T0L and T400L vs. T100L, respectively. The differential metabolites mainly enriched in metabolic pathways, secondary metabolite synthesis, carbon metabolism, ABC transport and various amino acid metabolism between T400L treatment and T0L treatment; and aspartic acid, fumaric acid, cysteine, oxaloacetic acid and proline were involved in 16, 10, 9, 9 and 6 metabolic pathways, respectively (Fig. 7; Table S3).

### Metabolic pathway analysis of differentially accumulating metabolites

**Figure 8:**
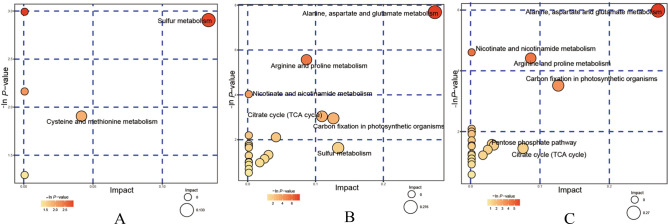
Pathway analysis of the leaves of *N. sibirica* in response to the different treaments. Each bubble in the bubble graph represents a metabolic pathway. The abscissa and size of the bubble represent the influencing factor of this pathway in the topological analysis; the larger the size is, the greater impact factor. The ordinate and color represent the *P*-value of the enrichment analysis (− ln[*P*-value]); the deeper the color is, the smaller the *P*-value and the more significant the degree of enrichment. (**A**) T0L vs. T100L; (**B**) T0L vs. T400L; (**C**) T100L vs. T400L.

KEGG annotation analyses reveal only differences in metabolites involved in pathways. To determine whether these pathways are closely associated with experimental conditions, further analysis of the metabolic pathways of the differentially accumulating metabolites is needed. The key pathway that exhibits the strongest correlation with metabolites can be found by comprehensively analyzing the differentially accumulating metabolites in the pathways (enrichment and topology analysis) and by further screening of the pathways^64^. The results of the metabolic pathway analysis are presented as a bubble plot (Fig. 8). Sulfur metabolism more strongly impact *N. sibirica* under the T100L treatment than under T0L treatment, whereas alanine, aspartate and glutamate metabolism; carbon fixation in photosynthetic organisms; and sulfur metabolism more strongly impact *N. sibirica* under T400L treatment. The comprehensive analysis of the metabolic pathways under the different treatments revealed that the differentially accumulating metabolites mapped to 26 pathways in the leaves. Alanine, aspartate and glutamate metabolism; the citrate cycle (TCA cycle); carbon fixation in photosynthetic organisms; and sulfur metabolism in the leaves are important metabolic pathways in response to salt stress (Table S4).

### Correlation analysis of DEGs and differential metabolites

**Figure 9:**
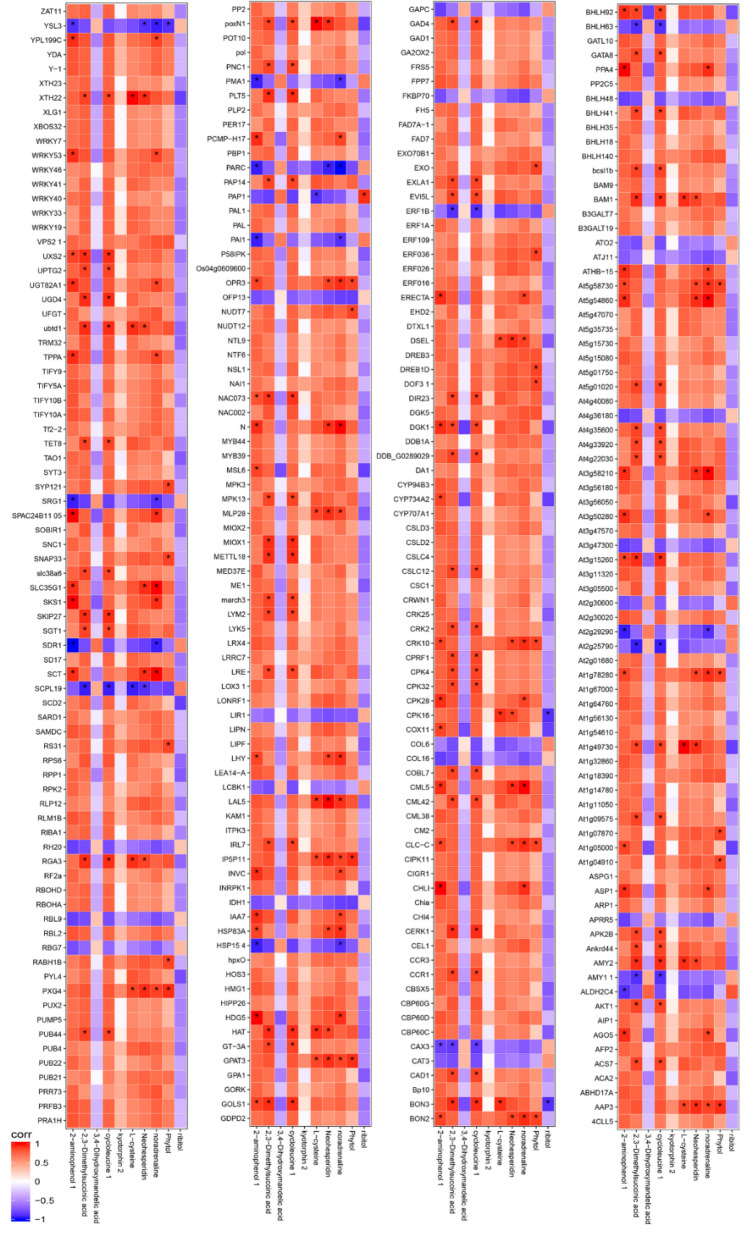
Heat map of correlation between differential genes and differential metabolites s in *N. sibirica* leaves (T100L vs T0L). *Indicates the correlation P value of differential metabolites and differential gene less than 0.05.

Through analysis of DEGs and differential metabolites between different treatments, 312 DEGs and 10 differential metabolites between T100L and T0L treatments had definitive annotation information. A total of 51 DEGs and 34 differential metabolites between the T400L and T0L treatments were identified in the annotation database. The Spearman algorithm determined the correlation coefficient (Corr) and P-value of the differential metabolites and DEGs, and the correlations are shown in Figs. 9 and S6. As shown in Fig. 9, between the T100L and T0L treatments, most of the differential metabolites were positively correlated with DEGs, while 3,4-dihydroxymandelic acid and phytol were negatively correlated with the DEGs. Moreover, 8 differential metabolites and 131 DEGs showed significant correlations (*P* < 0.05). Figure S6 shows that between the T400L and T0L treatments, the correlations of 25 differential metabolites and DEGs were positive, the correlations of 9 differential metabolites and DEGs were negative, and there were significant correlations between 18 differential metabolites and 51 DEGs (*P* < 0.05).

### Pathway analysis of DEGs and differential metabolites

To analyze in detail the relationship between DEGs and differential metabolites in leaves of *N. sibirica* under different NaCl treatments, the DEGs and differential metabolites between different treatments were mapped to the KEGG pathway data to construct an integrated map for the correlation analysis. As shown in Table S5, 47 DEGs and 3 differential metabolites between the T100L and T0L treatments were mapped to 26 metabolic pathways. The DEG-enriched metabolic pathways mainly included biosynthesis of secondary metabolites, plant pathogen interaction, mitogen-activated protein kinase (*MAPK*) signaling pathway, and amino acid metabolism, the metabolic pathways mapped by differential metabolites mainly included amino acid metabolism, and the DEGs and differential metabolites mapped to metabolic pathways were mainly upregulated. A total of 3 DEGs and 18 differential metabolites between T400L and T0L treatments were mapped to 28 metabolic pathways; the number of DEGs mapped to the secondary metabolite biosynthetic pathway was the largest at 3, and the differential metabolites were mainly mapped to secondary metabolite biosynthesis, carbon metabolism, and amino acid metabolism pathways. Under both T400L and T100L treatments, cysteine and aspartic acid were involved in multiple metabolic pathways, and the aspartate aminotransferase gene (*ASP1*) was involved in multiple metabolic pathways, suggesting that *ASP1* has an important role in the response to NaCl in *N. sibirica*.

### Correlation network analysis of transcriptome and metabolome data

**Figure 10:**
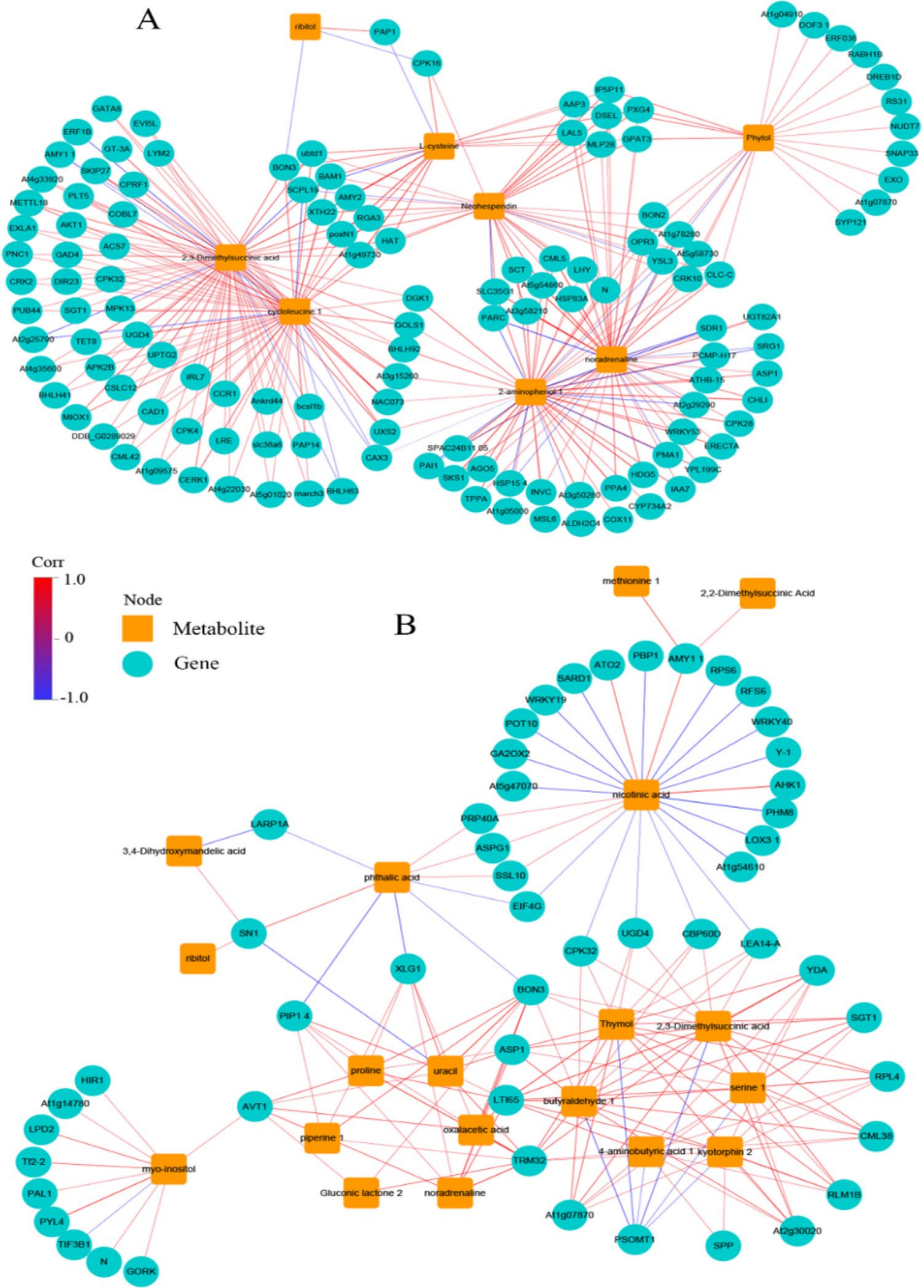
Differential genes-to-differential metabolites network in *N. sibirica* leaves. (**A**) T100L vs T0L; (**B**) T400L vs T0L. Orange squares represent differential metabolites, blue circles represent differential genes; connection colors represent correlations between nodes and nodes, red lines indicate positive correlations, and blue lines indicate negative correlations.

The correlation analysis of transcriptome and metabolome was performed for DEGs and differential metabolites screened according to |Corr|> 0.8 and *P* < 0.05. Through correlation screening, 131 DEGs and 8 differential metabolites were obtained between the T100L and T0L treatments. A total of 51 DEGs and 19 differential metabolites were obtained between the T400L and T0L treatments. The correlation network analysis is shown in Fig. 10. The analysis revealed that 18 DEGs and 8 differential metabolites between the T100L and T0L treatments were negatively correlated, and the remaining 113 DEGs and differential metabolites were positively correlated. A total of 25 DEGs and 11 differential metabolites between the T400L and T0L treatments were negatively correlated, and the synthesis of 8 differential metabolites, including ribitol, proline, methionine, and oxaloacetate, was only positively correlated with regulation of the DEGs. A comprehensive analysis showed that in *N. sibirica*, metabolites such as 2-aminophenol, chlorophyll, and l-cysteine, and DEGs such as *AMY2*, *BAM1*, and *GPAT3*, occupied important positions in the correlation network under T100L treatment, while under T400L treatment, metabolites such as 4-aminobutyric acid, butyraldehyde, proline, oxaloacetate, and uracil, and DEGs such as *ASP1, CML38, RPL4*, and *YDA*, played important roles in the correlation network.

Through a comprehensive analysis of metabolic pathways and correlation networks, it is found that under T100L treatment, α-amylase (*AMY2*), β-amylase (*BAM1*), glycerol-3-phosphate acyltransferase (*GPAT3*) and other genes expression increased, which increased the activity of amylase, thereby promoting the hydrolysis of starch at the metabolic level, which is conducive to the synthesis of downstream metabolites such as l-cysteine and 2-aminophenol. Under T400L treatment, the expression of aspartate aminotransferase gene (*ASP1*) is enhanced, which promotes the synthesis of amino acids and their derivatives; the up-regulated expression of calmodulin gene (*CML38*) can activate multiple stimulus signals to make stress-related gene expression and regulation synthesis of protein and metabolites.

## Discussion

### Physiological changes of *N. sibirica* in response to salt stress

By altering the contents of organic metabolites, including amino acids, proline, sugars, polyalcohols and so on, in their vacuoles under salt stress conditions, plants can reduce the water potential in their cytoplasm to maintain osmotic balance and prevent dehydration^6,65,66^. The osmotic substances (soluble sugars, proline and amino acids) and ABA contents differed in response to different treatment concentrations and at different times. The osmotic substances and ABA content significantly differed between the T400L treatment and the T0L treatment; no differences were observed between the T100L treatment and the T0L treatment. These results were consistent with those previously reported^53,57^ indicated that more osmotic substances need to be synthesized for osmotic adjustment under high NaCl concentrations and that the effects of low NaCl concentrations on *N. sibirica* osmotic stress were not significant.

### Transcriptional regulation of *N. sibirica* responses to salt stress

There are no whole-genome sequencing data available for *N. sibirica*, and the National Center for Biotechnology Information (NCBI) database contains only partial gene sequences related to salt stress, such as *NsNHX1, NtNHX1, NtSOS1,* and *NtP5CS*^55,57,58,67,68^. Here, we analyzed *N. sibirica* DEGS in response to salt treatment, and we found that the number of DEGs was significantly greater under low-concentration than under high-concentration salt treatment, which might be due to the strong salt tolerance of *N. sibirica* and the enhanced expression levels of a large number of genes under low-concentration salt treatment that can improve the adaptability of *N. sibirica* to salt stress. Concurrently, the number of upregulated genes was significantly greater than the number of down-regulated genes, potentially because NaCl treatment activates the expression levels of *N. sibirica* genes to promote more biological processes to improve salt tolerance.

There were relatively more DEGs in the GO terms of cellular processes, metabolic processes, catalytic activities, and cell wall, and DEGs in KEGG annotations were mainly involved in plant-pathogen interaction, carbon metabolism, various amino acid metabolism, alternative splicing, and plant signal transduction. The plant cell wall could prevent ions from entering protoplasts, thus playing an important role in defense and ion detoxification^69^. In the present study, the DEGs identified under the two different NaCl treatments were both significantly enriched in the GO term of cell wall, and the number of upregulated was significantly greater than the number of downregulated genes, indicating that *N. sibirica* may produce a defensive response through cell wall thickening in response to NaCl treatment. The cell wall simultaneously plays an important role in the activation of metabolic sites, which, when stimulated, can induce signal molecules related to mitogen MAPK and calmodulin^70,71^. A total of 5 genes related to MAPK1 were identified in response to T100L treatment. The effect of MAPK on plant resistance to stress is specific to plant and ion species, so its biological function still requires further analysis. Moreover, 30 and 7 unigenes related to calmodulin were identified under the T100L and T400L treatments, respectively. Calmodulin, as the second messenger of plant cell signal transduction, can regulate cell physiological metabolism and gene expression, and it can regulate ion transport under heavy metal stress^70^.

This study revealed that many DEGs of enzymes related to synthesis and degradation were enriched in metabolic processes involving proline, phenylalanine, tyrosine, tryptophan, alanine, and aspartic acid amino acid metabolism. Acetaldehyde dehydrogenase participates in the elimination of reactive oxygen species and catalyzes the oxidation of toxic aldehydes when plants are under stress, playing an important role in the regulation of antioxidant stress in plants^72^. 4 and 3 downregulated acetaldehyde dehydrogenase genes were identified under the T100L and T400L treatments, respectively. Expression of the acetaldehyde dehydrogenase gene in *Medicago sativa* L. also decreases in response to different concentrations of salt treatment, demonstrating the environmental adaptability of the plants^73^. Many DEGs of enzymes with roles in carbon metabolism processes were identified, such as the triose phosphate isomerase, trehalose-6-phosphate phosphatase, α-amylase, UDP glucose dehydrogenase, and triose phosphate isomerase genes. In addition, expression of the acetyl CoA-acyltransferase gene involved in organic acid and lipid metabolism was upregulated, while that of the acetyl CoA-synthetase gene was decreased. Simultaneously, the downregulated acetaldehyde dehydrogenase gene is also involved in this metabolic pathway. These results indicate that after NaCl treatment of *N. sibirica*, the metabolic pathways of organic metabolite synthesis and degradation are interrelated, and different genes coordinate with each other to maintain the osmotic balance.

### Metabolic responses of *N. sibirica* to salt stresses

The results of the PCA indicated that T400L treatment could significantly alter metabolite contents, while T100L treatment could not. Our previous physiological studies also proved that low salt concentration treatments have little effect on metabolite (organic osmolyte) contents in *N. sibirica*^52,74^; however, high salt concentration treatments can significantly alter metabolite contents^49,53^. This finding may be because *N. sibirica* is a halophyte, and as such, the species exhibits strong salt tolerance and is not affected by low salt concentration treatments.

The accumulation of amino acids can maintain cell membrane stability, improve osmotic adjustment and ultimately improve salt tolerance^75^. In this study, the amino acid metabolism increased in *N. sibirica* under T400L treatment, which led to the accumulation of 4-aminobutyric acid, serine, aspartic acid, and cysteine. Similar increases in amino acid contents in wild soybean, *Thellungiella salsuginea* and *A. lagopoides* have been reported^36,76,77^. The increase in amino acid contents may be due to the production of amino acids or to increased protein degradation induced by stress^78^. The increases of amino acid contents also may be those induced amino acids play a role in maintaining metabolic and osmotic homeostases during stress^79^. The variability in amino acid responses in different organs to different NaCl treatments indicates different roles of amino acids in those organs under saline conditions. As an osmoprotectant in plants, proline plays an important role in maintaining osmotic homeostasis^14,80^. Many studies have reported that proline accumulations have different functions in plants that are growing under adverse conditions, although the function of proline in plant stress adaptation mechanisms remains controversial^81^. In the present study, the proline contents significantly increased by more than onefold under T400L treatment. However, compared with those in response to the T0Ltreatment, the proline contents in the leaves in response to T100L treatment did not significantly change. The changes in proline contents in response to different treatments also confirmed the results of the PCA, as T100L treatment had little effect on the metabolic pathways. The differences in proline contents may be because *N. sibirica* is a dilute-salt halophyte; as such, in order to maintain the osmotic balance needed to accumulate proline in the leaves, resulting in higher osmotic pressure in the leaves. Metabolic profiling studies in barley have also proven that metabolites synthesized under salt stress are at least tissue and genotype specific^39^.

The accumulation of sugars in plants under stress are involved in osmotic regulation, carbon storage, and active oxygen radical scavenging^33^. The results showed that the contents of glucose and sucrose slightly decreased in the leaves and that the contents of fructose, xylose, galactose and mannose increased as the NaCl treatment concentration increased. The integrated analysis of glycometabolism revealed that the carbohydrate contents increased under saline conditions. Similar changes in carbohydrate content increases in *Thellungiella halophila*, *Limonium latifolium* and *Populus euphratica* have also been reported^82–84^. However, glucose contents in maize have been confirmed to decrease under salt stress^85^. The accumulation of carbohydrates in plants plays an important role in maintaining osmotic balance, stabilizing macromolecules and providing available energy for plants to resume growth after salt stress^78^. In our study, the degradation of glucose and sucrose increased under stress, as more substrates were generated for other metabolic pathways. This response suggests that the production of downstream metabolites of metabolic flux is essential for plant salt tolerance.

The regulation of organic acid metabolism is an effective means for plants to adapt to salt stress^86,87^. We found that the contents of intermediate metabolites, including oxaloacetate, malate, fumarate, citrate and succinate, increased in the leaves under the T400L treatment, promoting the TCA cycle. Thus, plants such as wild soybean, *Poa pratensis,* and *N. sibirica* exhibit enhanced salt tolerance by increasing their TCA cycle production to improve both energy capacity and intermediate metabolite levels^76,88^. Enhanced TCA cycle production associated with glycolysis in the leaves will release more energy and accelerate physiological metabolic reactions, which is a basic response of plants with respect to stress tolerance.

### Gene-to-metabolite networks of *N. sibirica* in response to salt stresses

Through correlation analysis of the transcriptome and metabolome, we determined the correlation between DEGs and differential metabolites, the pathways involving the DEGs and differential metabolites, and the correlation network of the transcriptome and metabolome in response to different treatments.

The interaction between DEGs and differential metabolites in the leaves of *N. sibirica* can be clearly explained by the mapped KEGG metabolic pathways. The main enriched metabolic pathways for both T100L and T0L treatments included the biosynthesis of secondary metabolites, plant pathogen interaction, MAPK signaling pathway, and various amino acid metabolism. Analysis of transcription showed that plant pathogen interaction, MAPK signaling pathway, and amino acid metabolism played important roles in plant resistance to stress. Many scholars have found that NaCl treatment increased the contents of secondary metabolites such as ginsenosides in ginseng and glycyrrhizic acid in licorice^89,90^. The related DEGs and differential metabolites enriched in these metabolic pathways were mainly upregulated in the present study, which enhanced the adaptability of *N. sibirica* to NaCl treatment.

The analysis revealed that the differential metabolites, which were highly correlated with the DEGs, were positively correlated, and the contents of some metabolites, such as organic acids, amino acids, and polyhydric alcohols, increased under the coordinated control of multiple genes, for example, oxaloacetate, 4-aminobutyric acid, and proline. Increased organic acid contents in plants under stress play an important role in regulating the ion balance and pH^91^. Under salt stress, the accumulation in plants of small molecules such as amino acids are conducive to osmotic and metabolic regulation and has an important role in reducing plant damage caused by salt stress^92^. The 4-aminobutyric acid protects the thylakoid membrane from damage, reduces active oxygen-induced damage, and regulates signal transduction under stress. Moreover, it also plays an important role in glutamate metabolism in plants^93,94^. Physiological and metabolomics studies have shown that proline has an important role in resisting adverse environments, and the relative content of proline was highest following the different treatments. Correlation network analysis revealed 7 DEGs that are involved in the regulation of proline metabolism, including aspartic protease (*ASP1*), vacuolar amino acid transporter (*AVT1*), low temperature-induced protein (*LTI65*), and guanine nucleotide binding protein (*XLG1*), among others, indicating that NaCl treatment induces the expression of genes related to the proline synthesis pathway. Simultaneously, the expression levels of stress- and transport-related proteins were enhanced to directly or indirectly participate in the proline synthesis pathway and promote the accumulation of proline, thereby enhancing the resistance of *N. sibirica* to salt stress.

Combined analysis of metabolic pathways and correlation network showed that low-concentration salt treatment could increase the expression levels of the α-amylase (*AMY2*), β-amylase (*BAM1*), and glycerol-3-phosphate acyltransferase (*GPAT3*) genes at the upstream transcriptional regulation level, as well as increase the activity of amylases, thereby promoting the hydrolysis of starch at the metabolic level, which is beneficial to the synthesis of downstream metabolites such as, l-cysteine and 2-aminophenol. High-concentration salt treatment can enhance the expression of the aspartate aminotransferase gene (*ASP1*), promote the synthesis of amino acids and their derivatives, promote the upregulation of the calmodulin gene (*CML38*), activate multiple stimulus signals to promote the expression levels of stress-related genes and regulate the synthesis of proteases and metabolites, and at simultaneously enhance the expression of the ribosomal protein gene (*RPL4*) to promote the synthesis of proteins and enzymes.

## Conclusion

**Figure 11:**
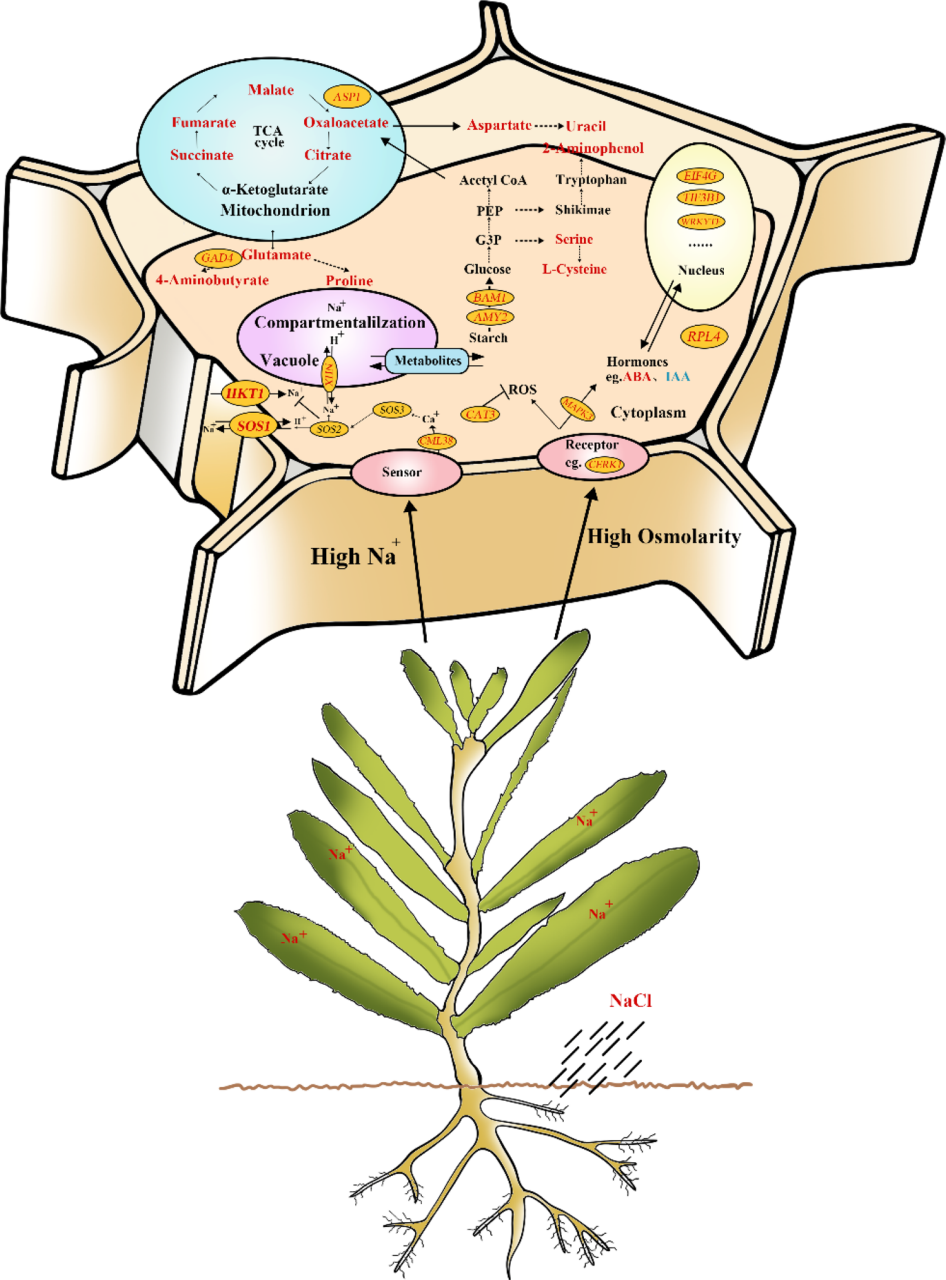
Diagram of the molecular and physiological metabolic pattern in response to salt stress in *N. sibirica.*

The molecular and physiological metabolic response mechanisms of *N. sibirica* to salt stress are shown^95^ in Fig. 11. After the NaCl treatments, a large amount of Na^+^ was absorbed by the roots and transferred to the leaves aboveground, resulting in an increase in Na^+^ accumulation in the leaves. The high Na^+^ concentration and high osmotic potential activated the plasma membrane-localized receptor kinase (*CERK1*) and promoted expression of *CML38* and *MAPK3* signal transduction genes, leading to ion efflux and compartmentalization to reduce the Na^+^ concentration in the cytoplasm. The enhanced expression of the catalase gene (*CAT3*) inhibited the increase in reactive oxygen species induced by osmotic stress. Moreover, salt stress-stimulating signals caused an increase in abscisic acid (ABA) and a decrease in indole-3-acetic acid (IAA) contents, which can promote the transcriptional expression of genes or transcription factors such as *EIF4G*, *TIF3BI* and *WRKY*. To maintain osmotic balance in the cells, starch was hydrolyzed by 2 amylases, *AMY2* and *BAM1*, and then the accumulation of 2-aminophenol and cysteine was promoted after a series of biochemical reactions. Simultaneously, the intermediate products entered the tricarboxylic acid (TCA) cycle, which promoted the TCA cycle and increased the expression of ASP1, inducing the accumulation of metabolites such as oxaloacetate and downstream products of the TCA cycle (4-aminobutyric acid, proline, and uracil). Therefore, through the synergistic effects of signal transduction, ion transport, and genes related to metabolite synthesis, *N. sibirica* regulates the contents of ions and metabolites in cells to maintain the osmotic balance, which is an important molecular and physiological metabolic regulatory mechanism in response to salt stress. These results may provide useful data for understanding the salt tolerance mechanisms in *N. sibirica*, which would further facilitate the effective management of halophyte cultivation.

## Materials and methods

### *Nitraria sibirica* Pall. cultivation

*Nitraria sibirica* seeds were permitted to collect from the Keluke beach of experimental station of Qinghai Forestry Research Institute (Qaidam basin, Qinghai province, China). All the studies of *N. sibirica* were carried out in accordance with relevant experimental guidelines, national or international guidelines. The seeds were germinated and then selected for sowing into containers filled with vermiculite: perlite (3:1) in March of the following year. Two-month-old seedlings were transplanted to rectangular plastic containers (10 L) that received continuous aerated hydroponic solution. Tap water was used in the first stage until the seedlings generated new roots, and half-strength Hoagland nutrient solution was supplied thereafter. The hydroponic solution was renewed every 4 days, and the pH was maintained at 6.0. The seedlings were grown in the greenhouse at 25 ± 3 °C under 14-h days/20 ± 2 °C under 10-h nights^60^.

### Salt treatment and sampling

Salt treatment began 7 days after the seedlings were transplanted. NaCl at a concentration of 50 mM/day was added to avoid osmotic shock and to allow the plants to express their acclimation potential; treatments were initiated such that all treatments reached their target concentration of 100, 200, 300 or 400 mM at the same time. A hydroponic solution without NaCl served as the control. To measure the physiological indexes, namely, the soluble sugar, amino acid, proline and abscisic acid (ABA) contents, three replicates of *N. sibirica* leaves were sampled on the first, third, seventh and fourteenth days after treatment. Additionally, the leaves of *N. sibirica* that were harvested at 3 days after treatment were used for transcriptomics and metabolomics analysis, and there were 3 and 7 replicates of per treatment, respectively. All of the samples were harvested in the middle of the light period, as the majority of the detectable and quantifiable metabolites are subject to strong diurnal rhythm^96,97^. The samples were immediately frozen in liquid nitrogen and stored at − 80 °C until further use.

### Measurements of soluble sugar, proline, amino acid and ABA contents

The soluble sugar and proline contents were measured in accordance with the sulfuric acid-anthrone colorimetric method and the ninhydrin method, respectively^98,99^. The amino acid contents were determined using an amino acid content assay kit (Solarbio, Beijing, China) in accordance with the manufacturer’s protocol. The ABA contents were measured using an enzyme-linked immunosorbent assay (ELISA)^100^.

### Transcriptome analysis

On the basis of the *2.3* results, the *N. sibirica* seedlings at three days after being treated with 0, 100 and 400 mM NaCl were used for transcriptome and metabolomics analysis.

The analysis methods of transcriptome and qPCR were referred to the author's previous published articles^60^.

### Metabolite extraction and profiling analysis

Approximately 100 mg of frozen tissue was added to a 2-mL Eppendorf (EP) tube, after which 0.5 mL of extraction liquid (*V*_*methanol*_:*V*_*water*_ = 3:1) was added; 20 μL of ribitol (1 mg/mL stock in dH_2_O) was subsequently added as an internal standard. Each tube was then vortexed for 30 s, after which the sample was homogenized three times in a ball mill for 4 min at 45 Hz. Afterward, the sample was treated via ultrasound for 5 min (incubated in ice water); each tube was then centrifuged at 13,000 rpm for 15 min at 4 °C. The supernatant (0.35 mL) was subsequently transferred to a clean 2-mL GC–MS glass vial, after which 60 μL of each sample was removed and pooled as a quality control (QC) sample. The samples were then dried completely in a vacuum concentrator in the absence of heat. Afterward, 40 μL of methoxyamine hydrochloride (20 mg/mL in pyridine) was incubated at 80 °C for 30 min. Sixty microliters of N,O-bis-(trimethylsilyl) trifluoroacetamide (BSTFA) reagent (1% trimethylchlorosilane [TMCS], v/v) was added to the sample aliquots, after which the samples were incubated for 1.5 h at 70 °C. Ten microliters of a standard mixture of fatty acid methyl esters (FAMEs) (C8-C16:1 mg/mL; C18-C24:0.5 mg/mL in chloroform) was subsequently added to the QC sample during its cooling to room temperature. The samples were analyzed by a GC system coupled to a Pegasus HT TOF–MS system.

The GC-TOF–MS analysis was performed using an Agilent 7890 gas chromatograph system (Agilent Technologies, Santa Clara, CA, USA) coupled to a Pegasus HT TOF mass spectrometer. The system consisted of a DB-5 MS capillary column coated with 5% diphenyl cross-linked with 95% dimethylpolysiloxane (30 m × 250 μm inner diameter, 0.25 μm film thickness; J&W Scientific, Folsom, CA, USA). A 1-μL aliquot of each analyte was injected in splitless mode. Helium served as the carrier gas, the front inlet purge flow was 3 mL min^−1^, and the gas flow rate through the column was 1 mL min^−1^. The initial temperature was maintained at 50 °C for 1 min and then increased to 310 °C at a rate of 10 °C min^−1^, after which the temperature was maintained at 310 °C for 8 min. The injection, transfer line, and ion source temperatures were 280, 270, and 220 °C, respectively. The energy was -70 eV in electron impact mode. The MS data were acquired in full-scan mode and with an m/z range of 50–500 at a rate of 20 spectra/second after a solvent delay of 6.17 min^101^.

### Data preprocessing and metabolic pathway construction

The physiological index data were analyzed and mapped used Microsoft Excel 2016 (Redmond, WA, USA), SPSS 19.0 (Chicago, IL, USA) and Origin 9.0 (Northampton, MA, USA). The Chroma TOF 4.3X software of LECO Corporation and the LECO-Fiehn Rtx5 database were used for raw peak extractions, data baseline filtering and calibration, peak alignment, deconvolution analyses, peak identification and integration of peak areas^102^. Both the mass spectrum match and the retention index match were considered during metabolite identification. The peaks detected in < 50% of the QC samples or the peaks in the QC samples in which the relative standard deviation (RSD) > 30% were removed^103^.

Furthermore, data normalization, principal component analysis (PCA) and orthogonal projections to latent structures-discriminant analysis (OPLS-DA) were performed by the SIMCA 14.1 software package (MKS Data Analytics Solutions, Umea, Sweden) using the three-dimensional data, which included the peak number, sample name, and normalized peak area. The first principal component of variable importance in the projection (VIP) and the remaining variables were assessed by Student’s t-test (*P*-value > 0.05) for the differentially accumulating metabolites. In addition, the Kyoto Encyclopedia of Genes and Genomes (KEGG) (http://www.genome.jp/kegg/) and MetaboAnalyst (http://www.metaboanalyst.ca/) databases were used to search for the pathways of metabolites^39^. Metabolite analyses between the treatments and control were tested using Origin 9.0 (Northampton, MA, USA).

The correlation heat map was drawn according to Corr and P-value which were obtained by the spearman algorithm which was used to calculate the correlation between the differentially expressed genes in transcriptome and the differential metabolites in metabolome. Then, the differentially expressed genes and differentially expressed metabolites were mapped to the KEGG Pathway database simultaneously to obtain their common pathway information. Finally, differentially expressed genes and metabolites were screened based on the |Corr|> 0.8 and P < 0.05, and the correlation network diagram was constructed by using Cytoscape v3.7.1^104^ software.

## Supplementary Information

Supplementary Figures.

Supplementary Table S1.

Supplementary Table S2.

Supplementary Table S3.

Supplementary Table S4.

Supplementary Table S5.

## Data Availability

The datasets supporting the conclusions of this research and materials used in this research are available by contacting with the corresponding author. The transcriptome raw data has been submitted to the NCBI with the ID GSE113246.

## Abbreviations

ABA: Abscisic acid
DEGs: Differentially expressed genes
FAO: Agriculture Organization of the United Nations
FTICR-MS: Fourier transform ion cyclotron resonance-mass spectrometry
GC–MS: Gas chromatography-mass spectrometry
GC-TOF–MS: Gas chromatography-time-of-flight-mass spectrometry
GO: Gene ontology
KEGG: Kyoto encyclopedia of genes and genomes
LC–MS: Liquid chromatography-mass spectrometry
NMR: Nuclear magnetic resonance
OPLS-DA: Orthogonal partial least squares-discriminant analysis
PCA: Principal component analysis
qPCR: Quantitative polymerase chain reaction
ROS: Reactive oxygen species
TCA: Tricarboxylic acid cycle
TICs: Total ion chromatograms
VIP: Variable importance in project

## References

[1] FAO. In *Food and Agriculture Organization of the United Nations* (http://www.fao.org/3/a-i5499e.pdf, Rome, Italy 2016).

[2] FAO. In *FAO Land and Plant Nutricion Management Services* (http://www.fao.org/ag/agl/agll/spush, Rome, Italy, 2005).

[3] Zhang J, Zhang Y, Du Y, Chen S, Tang H (2011). Dynamic metabonomic responses of tobacco (Nicotiana tabacum) plants to salt stress. J. Proteome Res..

[4] Suzuki N, Koussevitzky S, Mittler R, Miller G (2012). ROS and redox signalling in the response of plants to abiotic stress. Plant Cell Environ..

[5] Yan K (2013). Physiological adaptive mechanisms of plants grown in saline soil and implications for sustainable saline agriculture in coastal zone. Acta Physiol. Plant..

[6] Munns R, Tester M (2008). Mechanisms of salinity tolerance. Annu. Rev. Plant Biol..

[7] Slama I, Abdelly C, Bouchereau A, Flowers T, Savouré A (2015). Diversity, distribution and roles of osmoprotective compounds accumulated in halophytes under abiotic stress. Ann. Bot..

[8] Chen TH, Murata N (2002). Enhancement of tolerance of abiotic stress by metabolic engineering of betaines and other compatible solutes. Curr. Opin. Plant Biol..

[9] Hare P, Cress W, Van Staden J (1998). Dissecting the roles of osmolyte accumulation during stress. Plant Cell Environ..

[10] Munns R (2005). Genes and salt tolerance: Bringing them together. New Phytol..

[11] Abebe T, Guenzi AC, Martin B, Cushman JC (2003). Tolerance of mannitol-accumulating transgenic wheat to water stress and salinity. Plant Physiol..

[12] Yan S, Tang Z, Su W, Sun W (2005). Proteomic analysis of salt stress-responsive proteins in rice root. Proteomics.

[13] Wu W (2008). The Suaeda liaotungensis kitag betaine aldehyde dehydrogenase gene improves salt tolerance of transgenic maize mediated with minimum linear length of DNA fragment. Euphytica.

[14] Liu J, Zhu J-K (1997). Proline accumulation and salt-stress-induced gene expression in a salt-hypersensitive mutant of Arabidopsis. Plant Physiol..

[15] Urano K, Kurihara Y, Seki M, Shinozaki K (2010). ‘Omics’ analyses of regulatory networks in plant abiotic stress responses. J Curr. Opin. Plant Biol..

[16] Sumner LW, Mendes P, Dixon RA (2003). Plant metabolomics: Large-scale phytochemistry in the functional genomics era. Phytochemistry.

[17] Gygi SP (1999). Quantitative analysis of complex protein mixtures using isotope-coded affinity tags. Nat. Biotechnol..

[18] Trick M, Long Y, Meng J, Bancroft I (2010). Single nucleotide polymorphism (SNP) discovery in the polyploid Brassica napus using Solexa transcriptome sequencing. Plant Biotechnol. J..

[19] Wang J (2015). Transcriptomic profiling of the salt-stress response in the halophyte Halogeton glomeratus. BMC Genom..

[20] Bahieldin A (2015). RNA-Seq analysis of the wild barley (*H. spontaneu*m) leaf transcriptome under salt stress. Comptes Rendus Biol..

[21] Li S (2016). Effects of drought and salt-stresses on gene expression in Caragana korshinskii seedlings revealed by RNA-seq. BMC Genom..

[22] Valliyodan B, Nguyen HT (2006). Understanding regulatory networks and engineering for enhanced drought tolerance in plants. Curr. Opin. Plant Biol..

[23] Yao D (2011). Transcriptome analysis reveals salt-stress-regulated biological processes and key pathways in roots of cotton (Gossypium hirsutum L.). Genomics.

[24] Barragán V (2012). Ion exchangers NHX1 and NHX2 mediate active potassium uptake into vacuoles to regulate cell turgor and stomatal function in Arabidopsis. Plant Cell.

[25] Hamamoto S (2015). HKT transporters mediate salt stress resistance in plants: From structure and function to the field. Curr. Opin. Biotechnol..

[26] Yang Y (2015). Overexpression of the PtSOS2 gene improves tolerance to salt stress in transgenic poplar plants. Plant Biotechnol. J..

[27] Aghaei K, Ehsanpour A, Shah A, Komatsu S (2009). Proteome analysis of soybean hypocotyl and root under salt stress. Amino Acids.

[28] Fan XD (2013). Gene expression profiling of soybean leaves and roots under salt, saline–alkali and drought stress by high-throughput Illumina sequencing. Gene.

[29] Ruan C-J, Teixeira da Silva JA (2011). Metabolomics: Creating new potentials for unraveling the mechanisms in response to salt and drought stress and for the biotechnological improvement of xero-halophytes. Crit. Rev. Biotechnol..

[30] Nicholson JK, Lindon JC, Holmes E (1999). 'Metabonomics': Understanding the metabolic responses of living systems to pathophysiological stimuli via multivariate statistical analysis of biological NMR spectroscopic data. Xenobiotica.

[31] Shulaev V, Cortes D, Miller G, Mittler R (2008). Metabolomics for plant stress response. Physiol. Plant..

[32] Kaplan F (2004). Exploring the temperature-stress metabolome of Arabidopsis. Plant Physiol..

[33] Kim JK, Bamba T, Harada K, Fukusaki E, Kobayashi A (2007). Time-course metabolic profiling in Arabidopsis thaliana cell cultures after salt stress treatment. J. Exp. Bot..

[34] Armengaud P (2009). Multilevel analysis of primary metabolism provides new insights into the role of potassium nutrition for glycolysis and nitrogen assimilation in Arabidopsis roots. Plant Physiol..

[35] Rizhsky L (2004). When defense pathways collide. The response of Arabidopsis to a combination of drought and heat stress. Plant Physiol..

[36] Sobhanian H, Motamed N, Jazii FR, Nakamura T, Komatsu S (2010). Salt stress induced differential proteome and metabolome response in the shoots of Aeluropus lagopoides (Poaceae), a halophyte C4 plant. J. Proteome Res..

[37] Jorge TF (2017). GC-TOF-MS analysis reveals salt stress-responsive primary metabolites in Casuarina glauca tissues. Metabolomics.

[38] Guo R (2015). Comparative metabolic responses and adaptive strategies of wheat (Triticum aestivum) to salt and alkali stress. BMC Plant Biol..

[39] Wu D (2013). Tissue metabolic responses to salt stress in wild and cultivated barley. PLoS ONE.

[40] Johnson HE, Broadhurst D, Goodacre R, Smith AR (2003). Metabolic fingerprinting of salt-stressed tomatoes. Phytochemistry.

[41] Zhang J, Yang D, Li M, Shi L (2016). Metabolic profiles reveal changes in wild and cultivated soybean seedling leaves under salt stress. PLoS ONE.

[42] Chen W (2013). A novel integrated method for large-scale detection, identification, and quantification of widely targeted metabolites: Application in the study of rice metabolomics. Mol. Plant.

[43] Rangan P, Subramani R, Kumar R, Singh AK, Singh R (2014). Recent advances in polyamine metabolism and abiotic stress tolerance. BioMed Res. Int..

[44] Shi H, Chan Z (2014). Improvement of plant abiotic stress tolerance through modulation of the polyamine pathway. J. Integr. Plant Biol..

[45] Kovács Z, Simon-Sarkadi L, Szűcs A, Kocsy G (2010). Differential effects of cold, osmotic stress and abscisic acid on polyamine accumulation in wheat. Amino Acids.

[46] Ni J (2015). Salinity-induced metabolic profile changes in Nitraria tangutorum Bobr. suspension cells. Plant Cell Tissue Org. Cult..

[47] Ni J, Wu X, Zhang H, Liu T, Zhang L (2012). Comparative analysis of salt tolerance of three nitraria species. For. Res..

[48] Li Q, Wang S, Xu J, Ren W, Zhao Y (2012). Comprehensive evaluation on salt tolerance of different desert shrubs in Ulan Buh Desert regions. Pratacult. Sci..

[49] Yang S, Zhang H-X, Liu T (2012). Effect of salt stress on osmotic adjustment substances in plants. For. Res..

[50] Zhang G (2013). Effects of iso-osmotic salt and water stresses on growth and ionic absorption and distribution in *Nitraria sibirica* seedlings. Agric. Res. Arid Areas.

[51] Sa R, Chen G (2013). Effect of exogenous spermidine on antioxidant enzyme system in leaves of Nitraria sibirica Pall. seedlings under salt stress. Acta Bot. Boreali-Occidentalia Sin..

[52] Cheng TL (2015). Comparison on osmotica accumulation of different salt-tolerant plants under salt stress. For. Res..

[53] Wu X, Ni J, Zhang H, Liu T, Zhang L (2012). Effects of salt stress on osmotic adjustment substances in three species of *Nitraria*. J. Northeast For. Univ..

[54] Wang L, Li F, Zhang W, Chen G, Lin X (2012). Isolation and characterization of Nitraria sibirica actin gene. Acta Pratacult. Sin.

[55] Wang L (2016). Isolation and characterization of a tonoplast Na^+^/H^+^ antiporter from the halophyte Nitraria sibirica. Biol. Plant..

[56] Xin T (2014). Isolation and Expression Analysis of a Vacuolar Membrane Na^+^/H^+^ Antiporter Gene NtNHX1 from Nitraria tangutorum. Sci. Silvae Sin..

[57] Zheng L (2014). Isolation and characterization of a Δ1-pyrroline-5-carboxylate synthetase (NtP5CS) from Nitraria tangutorum Bobr. and functional comparison with its Arabidopsis homologue. Mol. Biol. Rep..

[58] Zheng L, Gao Z, Wang J, Zhang H, Wang Y (2014). Molecular cloning and functional characterization of a novel CBL-interacting protein kinase NtCIPK2 in the halophyte Nitraria tangutorum. Genet. Mol. Res..

[59] Wang L, Li F, Zhang W, Chen G, Lin X (2012). Isolation and characterization of Nitraria sibirica actin gene. Acta Pratacult. Sin..

[60] Li H, Tang X, Zhu J, Yang X, ZDe Zhang H (2017). Novo transcriptome characterization, gene expression profiling and ionic responses of Nitraria sibirica Pall. under salt stress. Forests.

[61] Trygg J, Wold S (2002). Orthogonal projections to latent structures (O-PLS). J. Chemom..

[62] Kanehisa M, Sato Y, Kawashima M, Furumichi M, Tanabe M (2016). KEGG as a reference resource for gene and protein annotation. Nucleic Acids Res..

[63] Kanehisa M, Goto S (2000). KEGG: Kyoto encyclopedia of genes and genomes. Nucleic Acids Res..

[64] Xia J, Sinelnikov IV, Han B, Wishart DS (2015). MetaboAnalyst 30—making metabolomics more meaningful. Nucleic Acids Res..

[65] Shi D, Wang D (2005). Effects of various salt-alkaline mixed stresses on Aneurolepidium chinense (Trin) Kitag. Plant Soil.

[66] Yang C, Zhao N, Xu C, Liu B, Shi D (2012). Regulation of ion homeostasis in rice subjected to salt and alkali stresses. Aust. J. Crop Sci..

[67] Zheng L, Zhang H, He L, Wang Y (2013). Isolation and expression analysis of a plasma membrane Na+/H+ antiporter from Nitraria tangutorum. Acta Pratacul. Sin..

[68] Tang X (2014). Isolation and expression analysis of a vacuolar membrane Na+/H+ antiporter gene NtNHX1 from *Nitraria tangutorum*. Sci. Silvae Sin..

[69] Pourrut B, Shahid M, Dumat C, Winterton P, Pinelli E (2011). Lead uptake, toxicity, and detoxification in plants. Rev. Environ. Contam..

[70] DalCorso G, Farinati S, Furini A (2010). Regulatory networks of cadmium stress in plants. Plant Signal. Behav..

[71] Opdenakker K, Remans T, Vangronsveld J, Cuypers A (2012). Mitogen-activated protein (MAP) kinases in plant metal stress: Regulation and responses in comparison to other biotic and abiotic stresses. Int. J. Mol. Sci..

[72] Zhang H, Chen S, Li J, Zhang Y, Xu X (2010). Studies on Transformation of Aldehyde dehydrogenase (ALDH) into Tomato. Chin. Agric. Sci. Bull..

[73] Xu W, Wen Y, Ma X, Luo F, Ren J (2014). Expression of aldehyde Dehydrogenase (ALDH) gene of alfalfa under adversity stress. Grassl. Turf.

[74] Li H, Yang X, Tang X, Zhang H (2019). Effects of NaCl stress on main osmoregulation substance and hormones contents of *Nitraria sibirica* Pall. leaves. J. Northeast For. Univ..

[75] Widodo (2009). Metabolic responses to salt stress of barley (*Hordeum vulgare* L.) cultivars, Sahara and Clipper, which differ in salinity tolerance. J. Exp. Bot..

[76] Li M (2017). Comparison of Salt Tolerance in Soja Based on Metabolomics of Seedling Roots. Front. Plant Sci..

[77] Lugan R (2010). Metabolome and water homeostasis analysis of Thellungiella salsuginea suggests that dehydration tolerance is a key response to osmotic stress in this halophyte. Plant J..

[78] Llanes A, Arbona V, Gómez-Cadenas A, Luna V (2016). Metabolomic profiling of the halophyte Prosopis strombulifera shows sodium salt-specific response. Plant Physiol. Biochem..

[79] Quan R, Shang M, Zhang H, Zhao Y, Zhang J (2004). Improved chilling tolerance by transformation with betA gene for the enhancement of glycinebetaine synthesis in maize. Plant Sci..

[80] Delauney AJ, Verma DPS (1993). Proline biosynthesis and osmoregulation in plants. Plant J..

[81] Lehmann S, Funck D, Szabados L, Rentsch D (2010). Proline metabolism and transport in plant development. Amino Acids.

[82] Gagneul D (2007). A reassessment of the function of the so-called compatible solutes in the halophytic Plumbaginaceae Limonium latifolium. Plant Physiol..

[83] Gong Q, Li P, Ma S, Indu Rupassara S, Bohnert HJ (2005). Salinity stress adaptation competence in the extremophile Thellungiella halophila in comparison with its relative Arabidopsis thaliana. Plant J..

[84] Brosché M (2005). Gene expression and metabolite profiling of Populus euphratica growing in the Negev desert. Genome Biol..

[85] Gavaghan CL (2011). Application of NMR-based metabolomics to the investigation of salt stress in maize (Zea mays). Phytochem. Anal..

[86] Yang C (2007). Osmotic adjustment and ion balance traits of an alkali resistant halophyte Kochia sieversiana during adaptation to salt and alkali conditions. Plant Soil.

[87] Shi D, Sheng Y (2005). Effect of various salt–alkaline mixed stress conditions on sunflower seedlings and analysis of their stress factors. Environ. Exp. Bot..

[88] Hu L, Zhang P, Jiang Y, Fu J (2015). Metabolomic analysis revealed differential adaptation to salinity and alkalinity stress in Kentucky bluegrass (Poa pratensis). Plant Mol. Biol. Rep..

[89] Jeong GTA, Park DHE (2006). Enhanced secondary metabolite biosynthesis by elicitation in transformed plant root system. Appl. Biochem. Biotechnol..

[90] Yang X, Li J, Dong X, Duan L, Li Z (2006). Effects of exogenous glycyrrhizinic acid on the seedling growth, glycyrrhizinic acid content of roots and some physiological indexes of *Glycyrrhiza uralensis* fisch seedling under NaCl stress. Plant Physiol. Commun..

[91] Shi D, Yin S, Yang G, Zhao K (2002). Citric acid accumulation in an alkali-tolerant plant *Puccinellia tenuiflora* under alkaline stress. Acta Bot. Sin..

[92] Munns R (2010). Comparative physiology of salt and water stress. Plant Cell Environ..

[93] Bouché N, Fromm H (2004). GABA in plants: Just a metabolite?. Trends Plant Sci..

[94] Billinton A (2001). Advances in the molecular understanding of GABAB receptors. Trends Neurosci..

[95] Taiz L, Zeiger E (2010). Plant Physiology.

[96] Urbanczyk-Wochniak E (2005). Profiling of diurnal patterns of metabolite and transcript abundance in potato (Solanum tuberosum) leaves. Planta.

[97] Lisec J, Schauer N, Kopka J, Willmitzer L, Fernie AR (2006). Gas chromatography mass spectrometry-based metabolite profiling in plants. Nat. Protoc..

[98] Bates L, Waldren R, Teare I (1973). Rapid determination of free proline for water-stress studies. Plant Soil.

[99] Spiro RG (1966). [1] Analysis of sugars found in glycoproteins. Methods Enzymol..

[100] Wu S, Chen W, Zhou X (1988). Enzyme linked immunosorbent assay for endogenous plant hormones. Plant Physiol. Commun..

[101] Wang Y (2015). Metabolomic analysis with GC-MS to reveal potential metabolites and biological pathways involved in Pb & Cd stress response of radish roots. Sci. Rep..

[102] Kind T (2009). FiehnLib: Mass spectral and retention index libraries for metabolomics based on quadrupole and time-of-flight gas chromatography/mass spectrometry. Anal. Chem..

[103] Dunn WB (2011). Procedures for large-scale metabolic profiling of serum and plasma using gas chromatography and liquid chromatography coupled to mass spectrometry. Nat. Protoc..

[104] Shannon P (2003). Cytoscape: A software environment for integrated models of biomolecular interaction networks. Genome Res..

