# Supplementary material for: Comprehensive transcriptome and metabolome profiling reveal metabolic mechanisms of Nitraria sibirica Pall. to salt stress
Source: Sci Rep. 2021 Jun 18;11:12878. doi: 10.1038/s41598-021-92317-6 (PMC8213879; doi:10.1038/s41598-021-92317-6)
Supplement: Supplementary file 1 — Supplementary Figures. [file 41598_2021_92317_MOESM1_ESM.docx]

**SUPPLEMENTARY INFORMATION**

**Title:**

Comprehensive transcriptome and metabolome profiling reveal metabolic mechanisms of *Nitraria sibirica* Pall. to salt stress

**Authors:**

Huanyong Li, Xiaoqian Tang, Xiuyan Yang, Huaxin Zhang


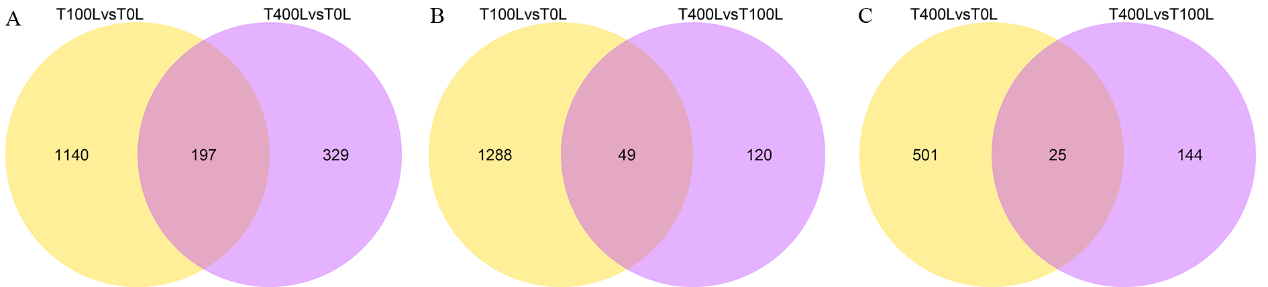


**Figure S1**. Venn diagrams of DEGs in *N. sibirica* treated with different concentrations of NaCl

**Figure S2**. Main metabolic pathways in the KEGG enrichment analysis of DEGs under different NaCl treatments. A indicate T100L vs. T0L; B indicate T400L vs. T0L; C indicate T400L vs. T100L.


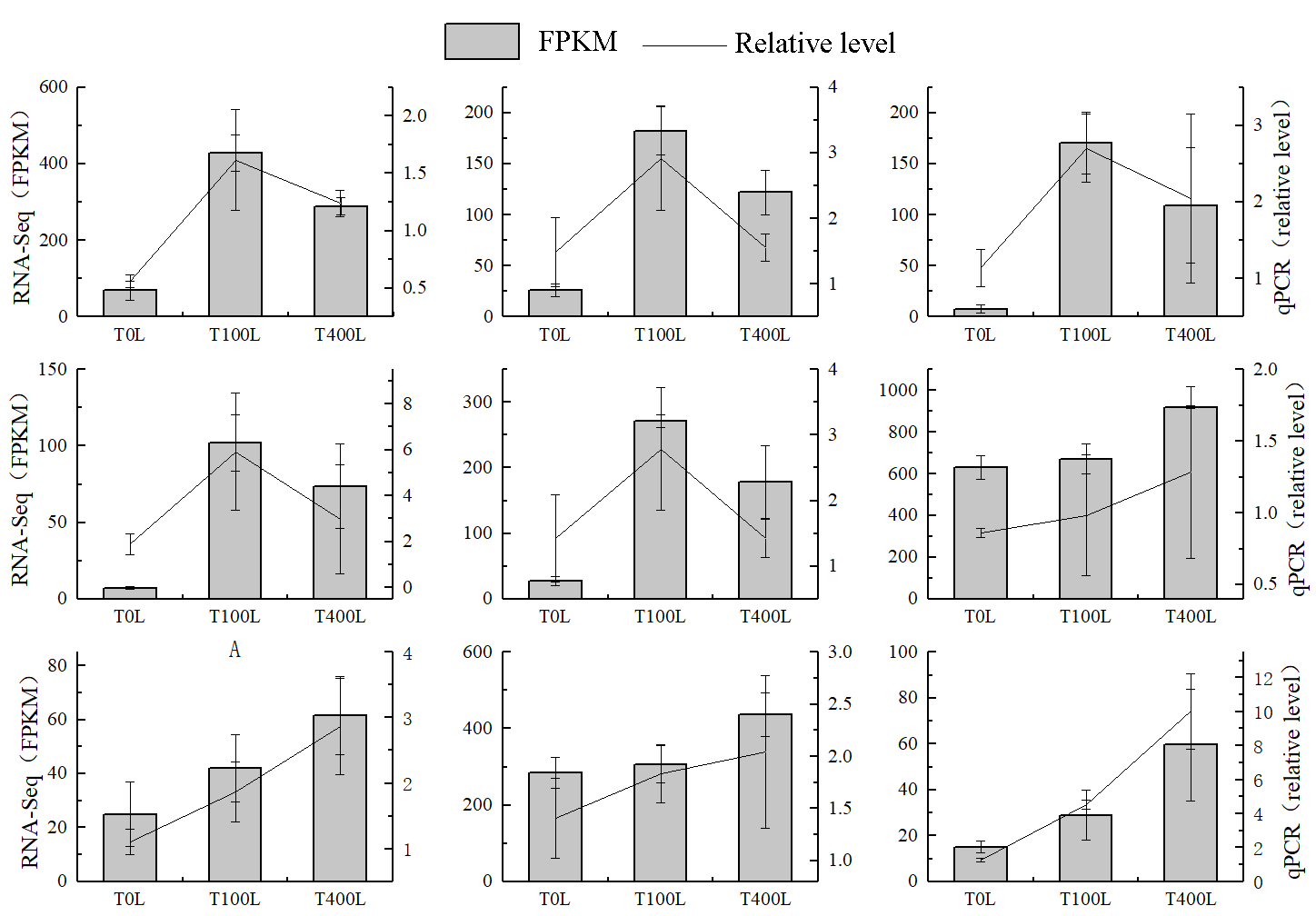


**Figure S3.** qPCR verification of unigene expression levels in *N. sibirica*


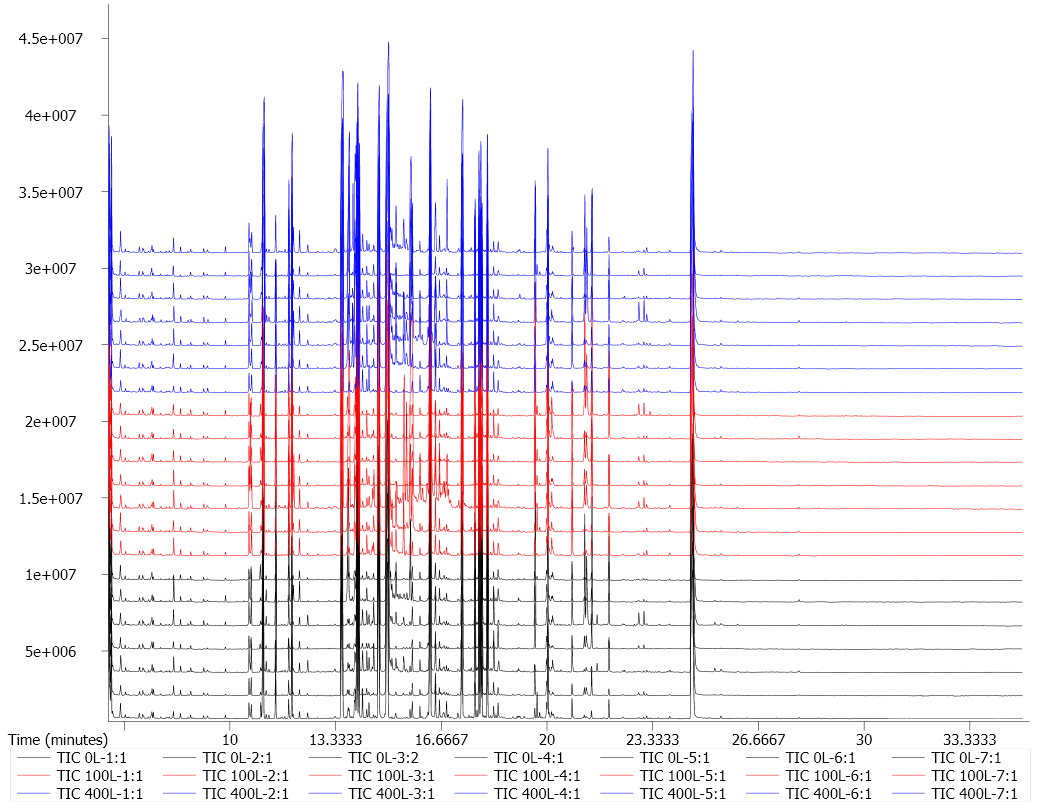


**Figure S4.** Total ion chromatogram（TIC）of *N. sibirica* leaves under different NaCl concentration treatment


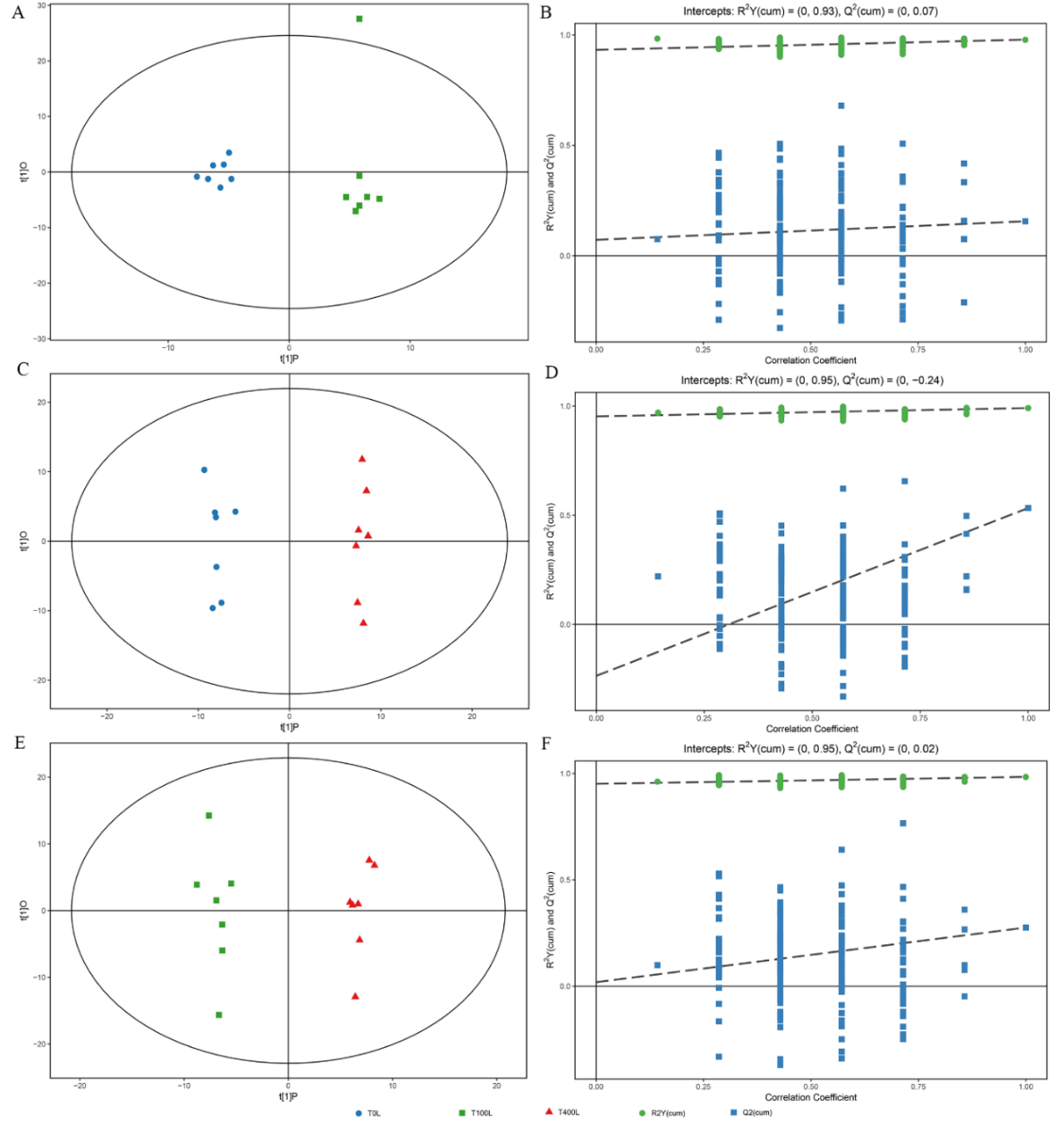


**Figure S5.** Score scatter plot and Permutation test of OPLS-DA model for metabolites in *N. sibirica* leaves under different NaCl concentration treatment. **A** and **B** indicate T100L vs. T0L; **C** and **D** indicate T400L vs. T0L; **E** and **F** indicate T400L vs. T100L


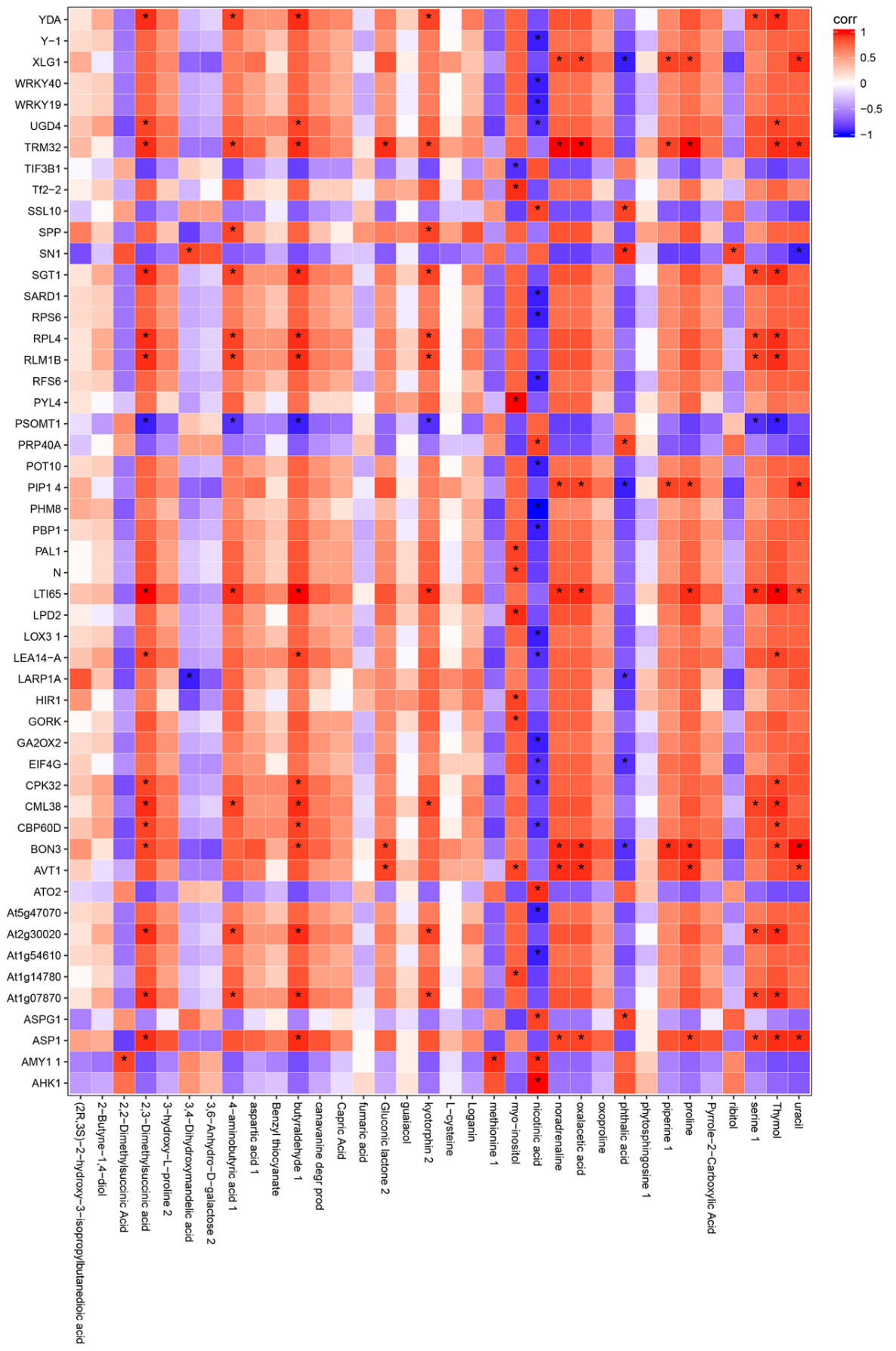


**Figure S6**. Heat map of correlation between differential genes and differential metabolites s in *N. sibirica* leaves (T400L vs T0L). Note: * indicates the correlation P value of differential metabolites and differential gene less than 0.05.
